# The role of extreme heat exposure on premature rupture of membranes in Southern California: A study from a large pregnancy cohort

**DOI:** 10.1016/j.envint.2023.107824

**Published:** 2023-02-13

**Authors:** Anqi Jiao, Yi Sun, David A. Sacks, Chantal Avila, Vicki Chiu, John Molitor, Jiu-Chiuan Chen, Kelly T Sanders, John T Abatzoglou, Jeff Slezak, Tarik Benmarhnia, Darios Getahun, Jun Wu

**Affiliations:** a Department of Environmental and Occupational Health, Program in Public Health, University of California, Irvine, CA, USA; b Department of Research & Evaluation, Kaiser Permanente Southern California, Pasadena, CA, USA; c Department of Obstetrics and Gynecology, University of Southern California, Keck School of Medicine, Los Angeles, CA, USA; d College of Public Health and Human Sciences, Oregon State University, Corvallis, OR 97331, USA; e Department of Population and Public Health Sciences, University of Southern California, Los Angeles, CA 90033, USA; f Department of Civil and Environmental Engineering, University of Southern California, CA, USA; g School of Engineering, University of California, Merced, USA; h Scripps Institution of Oceanography, University of California, San Diego, 9500 Gilman Drive #0725, CA La Jolla 92093, USA; i Department of Health Systems Science, Kaiser Permanente Bernard J. Tyson School of Medicine, Pasadena, CA, USA; j Institute of Medical Information, Chinese Academy of Medical Sciences and Peking Union Medical College, Beijing, China

**Keywords:** Premature rupture of membranes, Heatwave, Temperature, Air pollution, Green space, Smoking

## Abstract

**Background:**

Significant mortality and morbidity in pregnant women and their offspring are linked to premature rupture of membranes (PROM). Epidemiological evidence for heat-related PROM risk is extremely limited. We investigated associations between acute heatwave exposure and spontaneous PROM.

**Methods:**

We conducted this retrospective cohort study among mothers in Kaiser Permanente Southern California who experienced membrane ruptures during the warm season (May-September) from 2008 to 2018. Twelve definitions of heatwaves with different cut-off percentiles (75th, 90th, 95th, and 98th) and durations (≥ 2, 3, and 4 consecutive days) were developed using the daily maximum heat index, which incorporates both daily maximum temperature and minimum relative humidity in the last gestational week. Cox proportional hazards models were fitted separately for spontaneous PROM, term PROM (TPROM), and preterm PROM (PPROM) with zip codes as the random effect and gestational week as the temporal unit. Effect modification by air pollution (i. e., PM_2.5_ and NO_2_), climate adaptation measures (i.e., green space and air conditioning [AC] penetration), sociodemographic factors, and smoking behavior was examined.

**Results:**

In total, we included 190,767 subjects with 16,490 (8.6%) spontaneous PROMs. We identified a 9–14% increase in PROM risks associated with less intense heatwaves. Similar patterns as PROM were found for TPROM and PPROM. The heat-related PROM risks were greater among mothers exposed to a higher level of PM_2.5_ during pregnancy, under 25 years old, with lower education and household income level, and who smoked. Even though climate adaptation factors were not statistically significant effect modifiers, mothers living with lower green space or lower AC penetration were at consistently higher heat-related PROM risks compared to their counterparts.

**Conclusion:**

Using a rich and high-quality clinical database, we detected harmful heat exposure for spontaneous PROM in preterm and term deliveries. Some subgroups with specific characteristics were more susceptible to heat-related PROM risk.

## 1. Introduction

Premature rupture of membranes (PROM), a common obstetrical problem with multifactorial etiology, refers to a rupture of the chorioamniotic membranes before the onset of labor (Duff, 1996; 1998; Wallace et al., 2016). PROM occurring after 37 gestational weeks is called term PROM (TPROM), accounting for more than 60% of PROM cases and affecting 8–10% of all pregnancies (Duff, 1996; Ha et al., 2018). Following TPROM, most women will experience the spontaneous onset of labor within 24 hours (Ismail and Lahiri, 2013; Middleton et al., 2017; Parry and Strauss, 1998). Preterm PROM (PPROM) describes PROM before 37 gestational weeks, which affects 3% of pregnancies or more than 120,000 pregnancies annually in the United States (Assefa et al., 2018; Menon and Richardson, 2017; Mercer, 2003). PPROM is linked to 30–50% of preterm births, as about 50–60% of women with PPROM remote from the term will deliver within one week even with conservative management (Lyons and McLaughlin, 2020; Mercer, 2003). PROM can significantly increase morbidity and mortality of both mothers and infants (Gonzalez-Mesa et al., 2021; Henderson et al., 2012; Mercer, 2003; Newman et al., 2009; Stan et al., 2013) and result in long-term neurodevelopment and mental health problems of infants (Clark and Varner, 2011; Patkai et al., 2013).

The causes of PROM are not well understood. PROM can result from the combined effects of various pathologic mechanisms as well as a physiologic weakening of the membranes, especially at full term (Caughey et al., 2008; Esteves, 2022; Richardson et al., 2020). Inflammation, oxidative stress, apoptosis, and activation of cytokines and matrix metalloproteinase may be the primary causes of PROM (Lannon et al., 2014; Romero et al., 2015). Bacterial products and pro-inflammatory cytokines likely change membrane morphology, while inflammatory mediators could damage fetal membrane integrity and activate uterine contractions (Esteves, 2022). From the public health perspectives, discovering modifiable risk factors is meaningful for targeted PROM prevention. Researchers have reported that environmental, behavioral, and sociodemographic factors, including bacterial/viral infections, low body mass index (BMI), lower socioeconomic status, and poor personal behaviors (e.g. smoking and using illicit drugs) (Assefa et al., 2018; Caughey et al., 2008; Lyons and McLaughlin, 2020; Menon and Richardson, 2017; Mercer, 2003), can contribute to higher PROM risks together with genetic susceptibility (Clark and Varner, 2011; Gonzalez-Mesa et al., 2021; Lannon et al., 2014; Tchirikov et al., 2018).

Exposure to extreme heat can result in dehydration, insufficient uterine blood flow, systematic inflammation, and oxidative stress (Carolan-Olah and Frankowska, 2014; Dadvand et al., 2011; He et al., 2016; McElroy et al., 2021; Stan et al., 2013; Sun et al., 2019; Yang et al., 2021), thereby leading to poor pregnancy outcomes including PROM (Ha et al., 2018; Song et al., 2019a). As yet, limited investigations have examined associations between acute exposure to high temperatures and PROM in which positive associations have been reported within the last gestational week (Ha et al., 2018; Song et al., 2019a; Yang et al., 2021). However, the duration of extremely hot days has not been considered in previous work. Due to climate change, the severity and duration of extreme heat events are increasing in certain regions, such as California (Ilango et al., 2020), making it urgent to study the adverse impact of heatwaves. So far, there has been no consistent definition of heatwaves (Huang et al., 2021) and some studies have highlighted the influence of atmospheric humidity on heat-related health impacts (Davis et al., 2016). Application of various heatwave definitions incorporating humidity is valuable for better capturing heat effects.

Maternal air pollution exposure has been identified as the potential environmental risk factor of PROM (Dadvand et al., 2014; Li et al., 2021a; Pereira et al., 2014; Pereira et al., 2016; Song et al., 2019b; Wallace et al., 2016; Wang et al., 2019; Zhang et al., 2021). Although results remain inconclusive, entire-pregnancy exposure to fine particulate matter < 2.5 μm (PM_2.5_) and nitrogen dioxide (NO_2_) were more commonly associated with elevated risks of PROM (Dadvand et al., 2014; Pereira et al., 2014; Wang et al., 2019). Associations between heatwaves and PROM might be modified by maternal exposure to air pollutants that can potentially amplify the vulnerability of people to high temperatures (Benmarhnia et al., 2014; Chen et al., 2018). In addition, given more frequent and intense heatwave events, it is important to understand whether climate adaptation factors such as green space and air conditioning (AC) use can modify the effects of heatwaves on adverse outcomes. Previous research has shown that green space reduced vulnerability to heat (Song et al., 2021), and that AC use helped prevent severe heat-related health risks (Romanello et al., 2021). Further, identifying susceptible subgroups with specific sociodemographic factors may inform the design of targeted population interventions (Jiao et al., 2019; Zhang et al., 2017).

This study assessed relationships between exposure to heatwaves during the final week of pregnancy and the risk of spontaneous PROM in a large pregnancy cohort from Southern California. Potential effect modification by air pollution, climate adaptation measures, and maternal factors was explored.

## 2. Methods

### 2.1. Study population

Approximately 340,000 pregnant women were included in our retrospective cohort from January 1st, 2008, to December 31st, 2018. The electronic health record (EHR) of each participant was collected from Kaiser Permanente Southern California (KPSC, CA, United States). KPSC, an integrated healthcare organization comprising 15 hospitals and 234 medical offices, provides care to about 4.6 million people in Southern California (Chen et al., 2019b). KPSC EHRs detail a wide range of data, including maternal demographic factors, residential histories during the entire pregnancy, medical records, self-reported health behaviors, and fetal characteristics. All maternal residential addresses were geocoded. We have detailed more information on this population in our prior work (Sun et al., 2021a). We restricted our population to mothers who experienced membrane ruptures during the warm season (May 1st-September 30th) to capture heat effects. To avoid PROM resulting from iatrogenic interventions, we excluded nonspontaneous PROM (n = 685, 4.0% of all PROM cases) in this study. In total, 190,767 subjects during the warm season in 2008–2018 were included in our analysis.

### 2.2. Health outcome measurements

The primary outcome was spontaneous PROM, the spontaneous ROM before the onset of labor regardless of gestational age (Pereira et al., 2014). The two subtypes of PROM (i.e., TPROM and PPROM) were examined as the secondary outcomes. PROM was diagnosed via a speculum vaginal examination by obstetricians in KPSC; pooling of fluid in the vagina or leakage from the cervix with an alkaline pH confirmed the diagnosis. To define the entire pregnancy, the date of conception was confirmed based on self-reported information about the last menstrual cycle and early pregnancy ultrasound (Sun et al., 2021a). The end of pregnancy in our study was truncated at the date of ROM (Dadvand et al., 2014). For mothers with PROM, the date of ROM was defined as the precise date of PROM events documented in KPSC EHRs. For mothers without PROM, we defined their dates of ROM as the delivery dates, since ROM normally occurs spontaneously during or at the beginning of labor (Dadvand et al., 2014).

### 2.3. Covariates

Potential covariates were a priori selected from the EHRs based on existing research (Assefa et al., 2018; Esteves, 2022; Ha et al., 2018). These included maternal age (< 25, 25–34, and ≥ 35, years), race/ethnicity (African American, Asian, Hispanic, non-Hispanic White, and multiple/others), educational level (lower than college, college, and higher than college), median family household income, pre-pregnancy BMI (kg/m^2^) [underweight (< 18.5), normal weight (18.5–24.9), overweight (25.0–29.9), and obese (≥ 30.0)], smoking status (non-smoker, ever smoker, and smoker during pregnancy), year of infant birth, and parity (primiparous and multiparous). Median family household income was obtained at the block-group level in 2013 (https://wonder.cdc.gov/).

### 2.4. Heatwave definitions

Heatwave effects can vary according to specific definitions and depend on local climate conditions (Xu et al., 2016). Informed by recent research (Ilango et al., 2020; Sun et al., 2020a), we combined four percentile thresholds of daily maximum heat index (HI) (i.e., 75th, 90th, 95th, and 98th) during the warm season from 2007 to 2018 in Southern California with consecutive durations of ≥ 2, ≥ 3, and ≥ 4 days to create 12 definitions of heatwaves. Daily maximum HI data at 4 km × 4 km grids based on the U.S. National Weather Service HI algorithm that incorporates daily maximum temperature and minimum relative humidity were derived from the outputs of gridMET datasets using both in-situ observational data and reanalysis (Abatzoglou, 2013; Dahl et al., 2019). Binary variables were generated for each heatwave definition, and participants were categorized as being in the exposed group when any heatwave event occurred during the last seven days of pregnancy.

### 2.5. Potential environmental effect modifiers

Entire-pregnancy exposure to PM_2.5_ and NO_2_ was primarily considered a susceptibility factor that may modify associations between heatwaves and PROM risks (Benmarhnia et al., 2014; Dadvand et al., 2014). Monthly averages of PM_2.5_ and NO_2_ were interpolated at a resolution of 200 m × 200 m with empirical Bayesian kriging (EBK) based on air quality measurements from monitoring stations of the U.S. Environmental Protection Agency. More details can be found elsewhere (Wu et al., 2016). Taking into account residential changes, exposure throughout the pregnancy was generated by averaging monthly data according to residential addresses of each participant.

Climate adaptation factors including green space and AC penetration were considered potential effect modifiers of heatwaves in our study. Green space data were derived from a machine learning model that we previously developed and evaluated (Sun et al., 2021b). Briefly, four-direction street view images at locations every 200 m of all the roads were obtained from Microsoft Bing Maps API. The amount of green space was estimated by averaging the proportion of greenery pixels in all street view images within the 200 m buffer zone for each residential location. Census tract-level AC penetration rates for the period spanning 2015 and 2016 were quantified based on hourly residential electricity records from Southern California Edison and daily average temperature data (Chen et al., 2019a). Populations living in climate zones with higher summertime temperatures tend to use AC more often (Chen et al., 2020). Therefore, we focused on climate zones where mothers experienced the most intense heatwave (i.e. daily maximum HI > 98th percentile lasting for ≥ 4 consecutive days) in the analysis of AC penetration.

### 2.6. Statistical analysis

To assess the acute effects of heatwave on spontaneous PROM during the final week of pregnancy, we fitted Cox proportional hazards (PH) models with gestational week as the temporal unit separately for each heatwave definition after adjusting for covariates. We checked the PH assumption for each model by adding time × covariate interaction terms, and stratified PH models were applied if any covariate violated the assumption (Bellera et al., 2010; Sheridan et al., 2019). Results were presented as hazard ratios (HRs) with 95% confidence intervals (CIs). ROMs occurring in October were censored if any day of the final gestational week was in September (Ilango et al., 2020). The zip code of maternal residence was added into the model as a random effect to control potential spatial clustering of PROM (Sun et al., 2020a). Separate analyses for sub-types of the outcome (i.e., TPROM and PPROM) were performed.

Stratified analyses of PROM were conducted to examine effect modification by exposure to air pollution (i.e., low [< 50th] vs high [≥ 50th] PM_2.5_ and NO_2_) during the entire pregnancy, climate adaptation measures (i.e., low [< 50th] vs high [≥ 50th] green space and AC penetration rate), demographic factors (i.e., age, race/ethnicity, education level, and income level), and maternal behavior (i.e., non-smoker vs smoker [including ever smoker and smoker during the pregnancy]) (Benmarhnia et al., 2014; Lyons and McLaughlin, 2020). The heterogeneity of subgroups was assessed using Cochran’s Q tests. In the sensitivity analysis, we further examined the effect modification by relatively shorter-term exposure to air pollution during the last three months and the last month of pregnancy. Moreover, interaction terms between every heatwave variable and every potential effect modifier were added into the models separately in the sensitivity analysis to further evaluate effect modification.

We performed sensitivity analyses for different exposure windows (four days vs two weeks before ROM), heatwave definitions based on daily maximum temperature (another commonly used temperature metric), and the adjustment of KPSC medical centers or county of residence as the random effect (Wallace et al., 2016). We also applied the discrete time approach with logit function and random effect of county as a sensitivity analysis (Murray et al., 2020). Gestational week was included as a covariate in a flexible manner (i.e. polynomials) in the model. We carried out our analyses with SAS version 9.4 (SAS institute, Cary, NC).

## 3. Results

The summary statistics of this study are shown in Table 1. From 2008 to 2018, 190,767 mothers who experienced membrane ruptures were identified in the warm season, including 16,490 cases of PROM (8.6%). Most of the mothers were between the ages of 25 and 34. Hispanic mothers accounted for about half of the total study population. About a third of the study cohort had an education level lower than college. More than 50% of mothers had a pre-pregnancy BMI value ≥ 25 kg/m^2^. About 5% of mothers smoked during the pregnancy. The average HI was 28.7 °C in the last gestational week for the total study population. More PROM cases were primiparous women. PPROM mothers were older (aged ≥ 35 years) and more likely to be obese and self-identify as African American or active smokers. Table S1 describes Pearson correlation coefficients between environmental factors. Overall, the average exposure level of HI during the last gestational week has a moderate correlation with the daily maximum temperature during the same exposure window (correlation coefficient r = 0.54), but little correlation with entire pregnancy exposure to air pollution (r = −0.004 for PM_2.5_ and r = −0.07 for NO_2_) and green space (r = 0.01). Average concentrations of entire-pregnancy PM_2.5_ and NO_2_ were moderately correlated with each other (r = 0.62). The exposure levels in the last three months and the last month of pregnancy were highly correlated with each other for both PM_2.5_ (r = 0.89) and NO_2_ (r = 0.90).

**Fig. 1.**
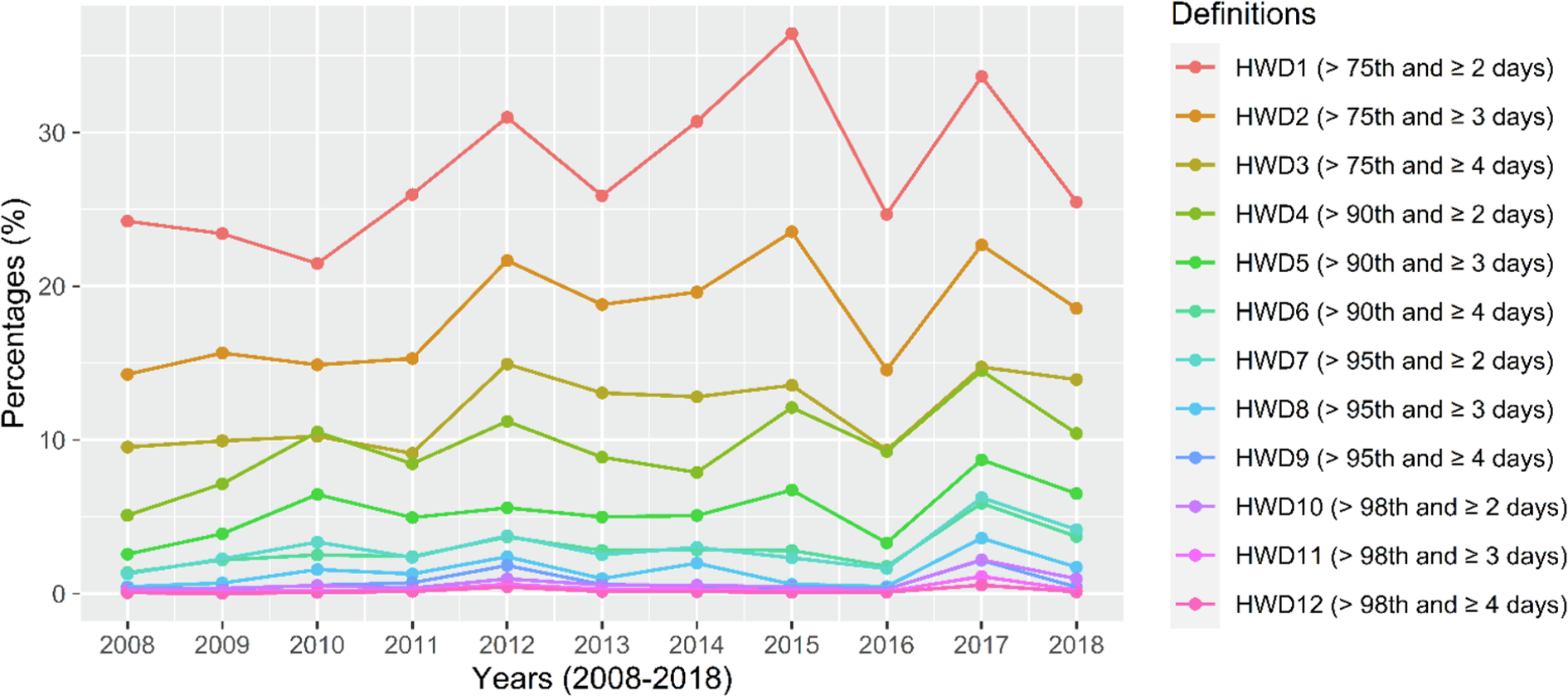
The yearly percentages of individual-experienced heatwave events for the total population in the warm season (May-September), 2008–2018. Heatwaves were defined by heat index thresholds (i.e., 75th, 90th, 95th, and 98th percentiles) and consecutive durations (i.e., lasting for ≥ 2 days [HWD1, HWD4, HWD7, and HWD10], ≥ 3 days [HWD2, HWD5, HWD8, and HWD11], and ≥ 4 days [HWD3, HWD6, HWD9, and HWD12]).

Table 2 presents the characteristics of 12 heatwave definitions (denoted as HWD1-HWD12) in this study. The number of mothers who suffered heatwaves using the least conservative definitions was 49,518 (26.0%) (HWD1: daily maximum HI > 32.8 °C lasting for ≥ 2 days), while only 339 (0.2%) participants experienced any heatwave event using the most conservative definitions (HWD12: daily maximum HI > 40.0 °C lasting for ≥ 4 days). About 1.8% of participants suffered two or more heatwave episodes of HWD1 in the last week of pregnancy. The number of PROM cases who experienced heatwaves ranged from 36 (0.2%) (HWD12) to 4,615 (28.0%) (HWD1), depending on the applied heatwave definition. Compared with the population without PROM, the percentages of mothers who experienced heatwaves were all slightly higher among PROM cases across 12 different definitions. The yearly percentages of mothers who experienced heatwave events roughly increased over time for most heatwave definitions; the largest percentage was found in 2015 (HWD1: 36.4%) (Fig. 1).

**Fig. 2.**
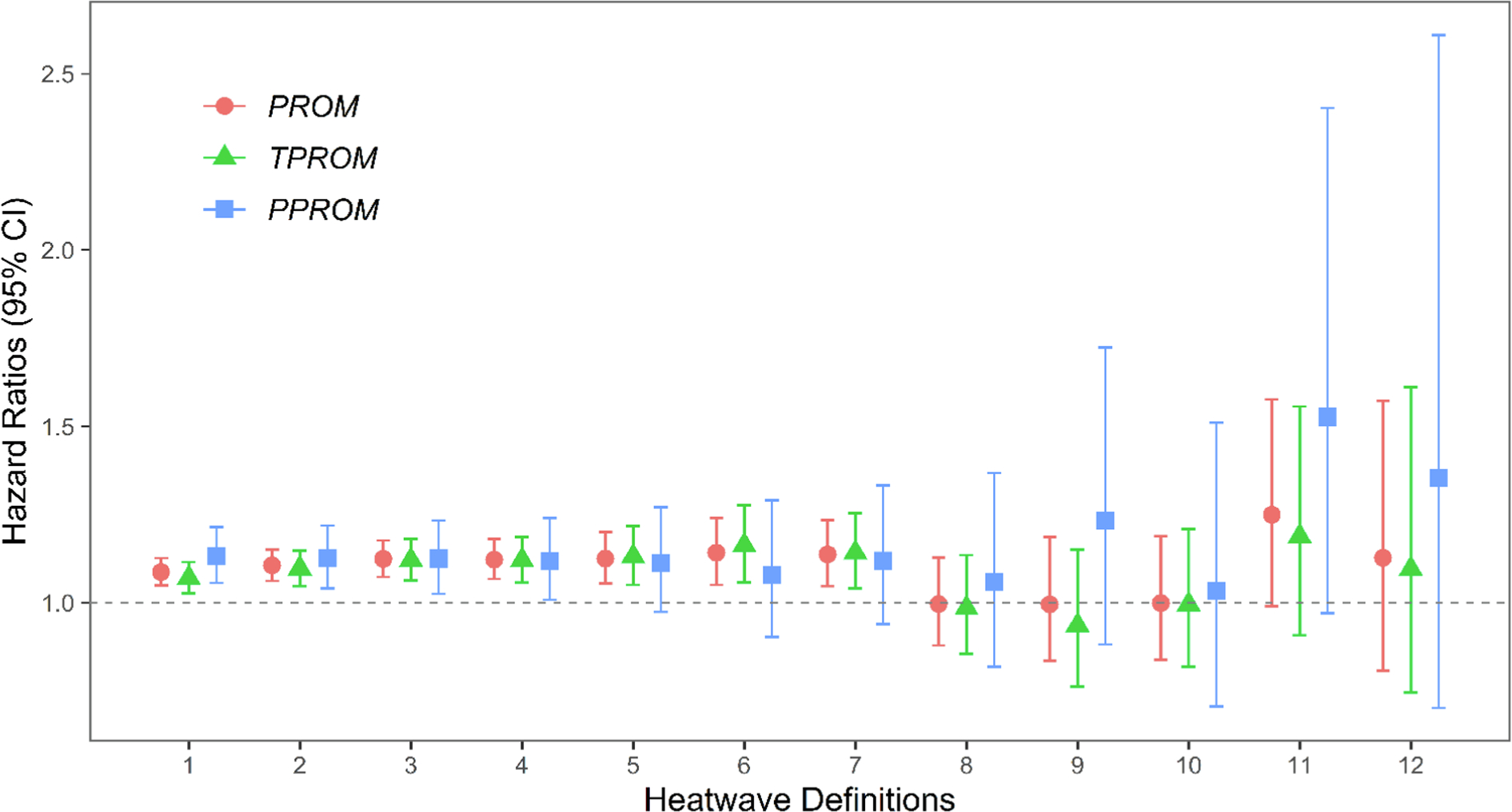
The estimated hazard ratios (HRs) with 95% confidence intervals (CIs) of PROM, TPROM, and PPROM associated with different heatwave definitions. Models are adjusted for maternal age, race/ethnicity, education level, median family household income, pre-pregnancy BMI, smoking status, year of infant birth, and parity.

As shown in Fig. 2, we found a 9–14% increase in the adjusted risk of PROM associated with HWD1-HWD7. Results of TPROM were similar to those of PROM with positive associations in HWD1-HWD7. Increased risks of PPROM were associated with less intense heatwave exposures (i. e., HWD1-HWD4). Wider CIs were observed for HWD9-HWD12, due to a much lower frequency of intense heatwave events and a smaller sample size of patients with PPROM. Details on the effect size and corresponding 95% CI for PROM, TPROM, and PPROM are provided in Table S2.

**Fig. 3.**
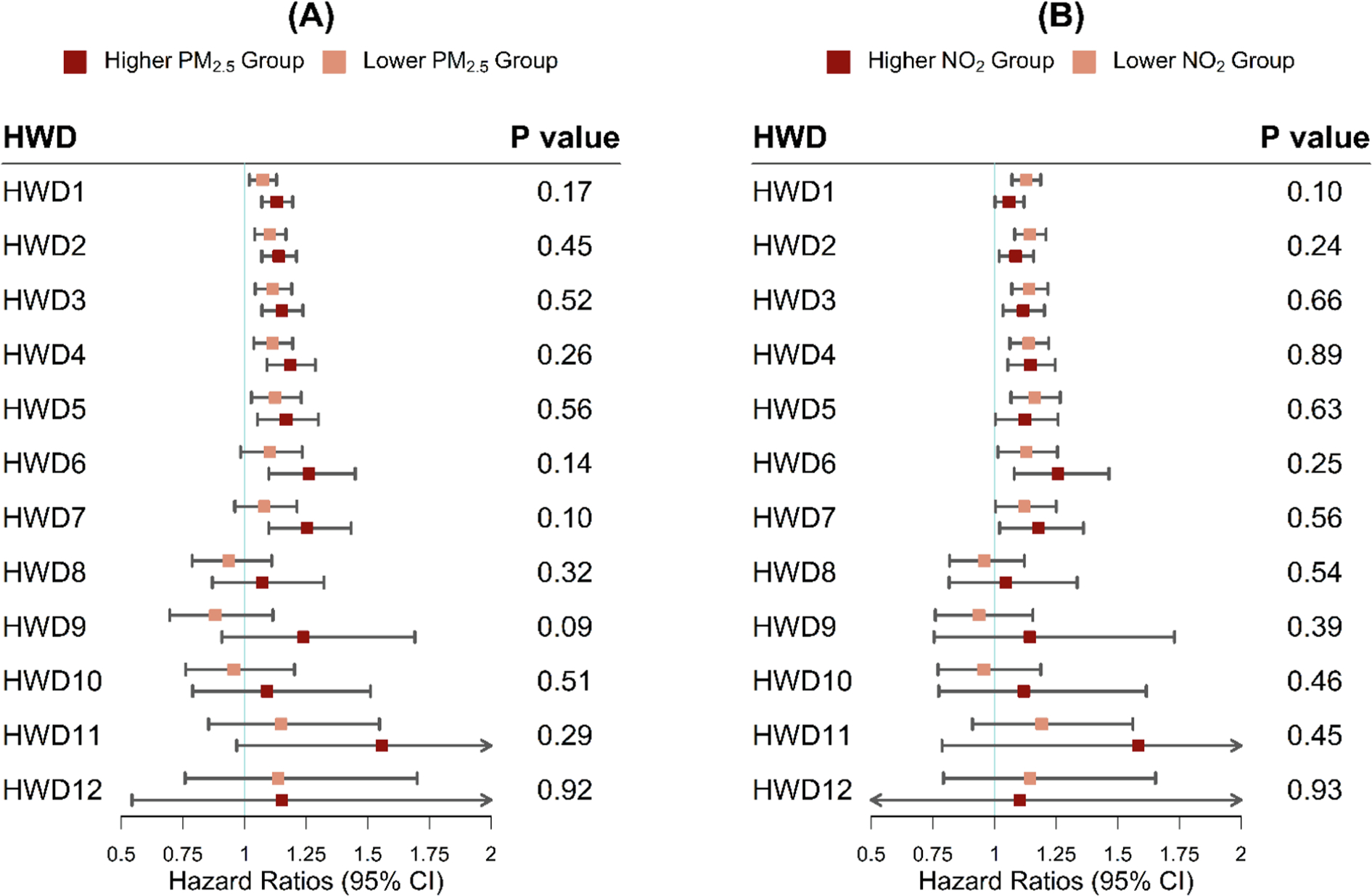
The hazard ratios (HRs) of PROM associated with different heatwave definitions in groups with lower (< 50th) vs higher (≥ 50th) entire-pregnancy exposure to PM_2.5_ (A) and NO_2_ (B). The cutoff concentrations of PM_2.5_ and NO_2_ are 11.26 μg/m^3^ and 16.45 ppb, respectively. Models are adjusted for maternal age, race/ethnicity, education level, median family household income, pre-pregnancy BMI, smoking status, year of infant birth, and parity. The *P* value refers to the comparison between subgroups and is obtained from Cochran’s Q test.

**Fig. 4.**
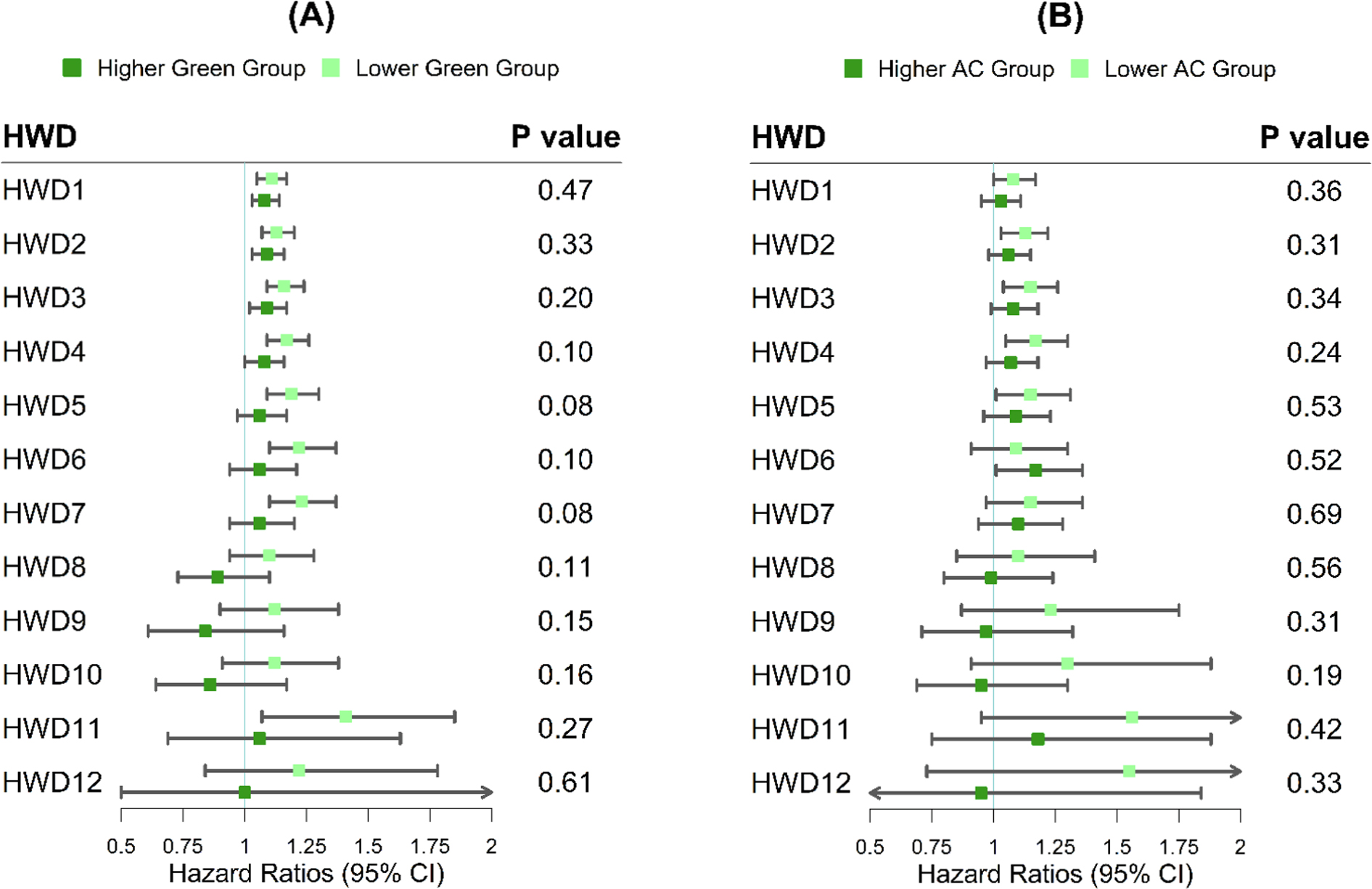
The hazard ratios (HRs) of PROM associated with different heatwave definitions in groups living in areas with lower (< 50th) vs higher (≥ 50th) levels of green space (A) and AC penetration rates (B). The cutoff points of subgroups are 24.24% and 82% for green space and AC penetration rates, respectively. The study areas in AC penetration analysis were restricted to climate zones where subjects experienced the most intense heatwave (i.e., HWD12). Models are adjusted for maternal age, race/ethnicity, education level, median family household income, pre-pregnancy BMI, smoking status, year of infant birth, and parity. The *P* value refers to the comparison between subgroups and is obtained from Cochran’s Q test. (For interpretation of the references to colour in this figure legend, the reader is referred to the web version of this article.)

Results of effect modification by air pollution exposure during the entire pregnancy and climate adaptation measures are presented in Fig. 3 (Table S3) and Fig. 4 (Table S6), respectively. Despite no significant heterogeneity detected by Cochran’s Q test, the observed associations between all the defined exposure to heatwaves and PROM risks were stronger among mothers who experienced higher whole-pregnancy exposure to PM_2.5_ (≥ 11.26 μg/m^3^). In the sensitivity analysis (Table S4–S5 and Figure S1–S2), we found significant effect modification by exposure to PM_2.5_ during the last three months of pregnancy, showing that mothers with greater PM_2.5_ exposure (≥ 10.98 μg/m^3^) had significantly higher risks of PROM associated with HWD1 and HWD2. The stratified analysis by climate adaptation measures showed that positive associations with a higher magnitude were identified among mothers living in areas with a lower level of residential green space or AC penetration rates.

Stratified analysis by demographic factors (Table S7) showed the risks of PROM associated with heatwave exposure were significantly higher in mothers under 25 years old (i.e., HWD2, HWD11, and HWD12). Most notably, we observed positive associations with a larger magnitude for the most intense heatwaves (HWD11: HR = 1.90, 95% CI: 1.30–2.80; HWD12: HR = 2.00, 95% CI: 1.24–3.25) among younger mothers. Results for different race/ethnicity groups showed no significant heterogeneity, even though positive associations were found in more heatwave categories among Hispanic and non-Hispanic White mothers. Further, some evidence suggests stronger associations between heatwaves and PROM in lower socioeconomic status groups. For all the heatwave categories, the positive associations between heatwaves and PROM were stronger among pregnant women with less education (vs those with a college degree or a higher education level), with some associations (HWD1-HWD4, HWD9, and HWD12) significantly different by education levels. The HR for HWD1 was also significantly higher in mothers with a lower income level. In terms of maternal behavior, mothers who smoked had a significantly higher risk compared to non-smokers (HWD4-HWD6).

In sensitivity analyses (Table S8), similar results were revealed for heatwaves defined four days or two weeks prior to the ROM, and stronger associations were observed with longer risk periods. Weaker associations were found with heatwave definitions based on daily maximum temperature compared with those based on HI in the main analyses. Controlling the random effect by the KPSC medical centers and county of residence changed the results minimally. Results derived from the discrete time approach were similar to the main model. The results of effect modification by air pollution exposure, climate adaptation measures, and maternal demographic and behavioral factors did not change substantially by performing models with the interaction term (Table S9–S13).

## 4. Discussion

We investigated associations between spontaneous PROM and heatwaves in a large pregnancy cohort in Southern California from 2008 to 2018 using rich and high-quality clinical data with accurate diagnosis dates and residential histories. Increased risk of spontaneous PROM was linked to acute heatwave exposure at the end of pregnancy. We provided evidence that higher exposure to PM_2.5_ during pregnancy, younger age, lower socioeconomic status, and smoking behavior were positive effect modifiers for heat-related PROM risks. Even though the potential climate adaptation factors in our study (i.e., green space and AC penetration) did not show statistically significant modification effects, subgroups living with lower green space or lower AC penetration had consistently higher PROM risks across heatwave categories compared to their counterparts; the effect modification by green space was marginally significant for some heatwave categories.

In this study, rather than applying ambient temperature as the exposure of interest, we considered a comprehensive list of heatwave definitions to account for both the intensity and duration of extremely high temperatures and incorporated the influence of atmospheric humidity by selecting HI as the temperature metric. We found consistent associations of increased risks of PROM with heatwave definitions of HWD1-HWD7. For the more intense heatwaves (i.e., HWD8-HWD12), we did not detect any effect on PROM, which may be due to lower frequencies of these extreme events resulting in reduced statistical power. This could also be explained by changes in behaviors or activity patterns of a population when facing extremely hot weather, such as reducing outdoor activities to offset adverse effects to some degree (Wang et al., 2020). Three previous investigations have explored associations of high temperatures with PROM, whose major conclusions were consistent with this study. A large nationwide case-crossover study from the United States was the first to associate elevated PROM risks with higher temperatures in the warm season (Ha et al., 2018). The authors found that for every 1 °C increase in temperature one week before delivery, the PROM risk increased by 4–5%. Two other studies conducted in China reported similar results. Increased PROM risks were associated with the 99th percentile of temperature relative to median temperature at lag 0–1, 0–2, 0–3 days (Song et al., 2019a), and with daily mean temperature higher than 30 °C relative to median temperature (18.7 °C) at lag 5, 6, 7 days (Yang et al., 2021). The latter two single-city studies both adopted the time-series design and used meteorological data based on very limited fixed-site monitoring stations, which can cause exposure misclassification due to the lack of individual exposure assessment and the ecological character of time-series studies. By applying maternal characteristics at the individual level and exposure measurement with a fine resolution, our findings based on mothers spanning 10 counties in Southern California add to the evidence for individual-level inferences about heat impacts on PROM. Moreover, we distinguished spontaneous PROM from all recorded cases based on the information provided by KPSC, which enabled us to provide a more accurate assessment of the outcome compared to previous studies.

We identified evidence of elevated risks associated with heatwaves across categories of PROM, though the estimated HRs for PPROM were more imprecise likely due to a relatively small number of PPROM cases (20.8% of the main PROM sample). Our results were in line with another study focusing mostly on the northeastern United States, which reported similarly increased risks of TPROM (odds ratio [OR] = 1.04, 95% CI: 1.03–1.05) and PPROM (OR = 1.05, 95% CI: 1.03–1.06) for every 1 °C increment in the average temperature during the week before delivery (during the warm season) (Ha et al., 2018). However, another time-series study in Shanghai, China, associated extremely high temperatures (90th and 97.5th percentiles vs 50th percentile temperature) only with TPROM, but not PPROM (Yang et al., 2021). One possible explanation is that the popularity of AC use in Shanghai may reduce the effect of extreme temperatures to some extent (Yang et al., 2021). Different demographic characteristics of study populations and study designs may further lead to heterogeneity in results. Given imprecise estimates with much wider CIs for extreme heat episodes (e.g. HWD8-HWD12) in this study, adverse effects of very extreme heatwaves on PROM and its subtypes merit further investigations for selecting appropriate heatwave definitions to develop regional heat warning systems and prevent poor pregnancy outcomes (Chen et al., 2015; Zhang et al., 2017).

To our knowledge, this study is the first to estimate how air pollution modifies the relationship between extreme heat and PROM. We found that maternal exposure to PM_2.5_ during the last three months of pregnancy positively modified the effects of heatwaves (i.e., HWD1 and HWD2). Higher heatwave-associated PROM risks were also observed among mothers with more severe exposure to PM_2.5_ throughout the pregnancy, with marginally significant heterogeneity between subgroups (i.e., HWD7 and HWD9 with *P* values ≤ 0.1). Our results suggest that acute heat effects might be more harmful to mothers chronically exposed to a higher level of ambient PM_2.5_ during pregnancy. Air pollution has been reported as the modifier of heat effects on mortality and morbidity, especially for respiratory and cardiovascular systems (Benmarhnia et al., 2014; Breitner et al., 2014; Ren et al., 2006). Although biological mechanisms of such effect modification may not be apparent for pregnancy outcomes, air pollution can increase the susceptibility to thermal effects by inducing circulating inflammation (Burkart et al., 2013). Therefore, a reduction in air pollution emissions may not only reduce air pollution-induced health problems but also mitigate heat-related adverse impacts. Furthermore, given that PM_2.5_ and NO_2_ are often released from the same sources (e.g., fossil-fuel combustion) as greenhouse gases, cutting air pollution emissions may have the associated benefit of reducing greenhouse gas emissions (West et al., 2013).

As global climate change has brought increasing challenges, it is crucial to inform possible adaptation strategies in reducing adverse health impacts of extreme heat. An increasing number of literature has reported benefits of higher green space exposure to mitigate urban warming problems and attenuate risks of morbidity and mortality (Burkart et al., 2016; Gronlund et al., 2015; Li et al., 2021b; Schinasi et al., 2018; Sun et al., 2020b; Xiao et al., 2018). When comparing the magnitude of heatwave effects on PROM among mothers with different green space exposure, we saw less positive associations for mothers with greater green space exposure, with marginally significant heterogeneity found in HWD4-HWD7 (*P* values ≤ 0.1), indicating that more surrounding green space might be a potentially protective modifier for heatwave exposure. More green plants and vegetation can not only enhance environmental comfort, but also improve mental health and life quality by enabling more contact with nature and increasing social cohesion (Beyer et al., 2014; Nutsford et al., 2013; Xiao et al., 2018). Future work might consider the direct relationships between green space exposure and PROM risks. Indoor cooling is another effective climate adaptation measure. A Lancet report on climate change demonstrated that indoor AC use prevented 195,000 heat-related deaths for older people (≥ 65 years) globally in 2019 (Bouchama et al., 2007; Romanello et al., 2021). To the best of our knowledge, existing research has not reported effect modification by AC use for heat effects on pregnancy outcomes. Based on AC penetration data at the census tract level in regions with higher summertime temperatures (Chen et al., 2020), we found a general trend showing higher risks of PROM due to heatwaves in census tracts with lower AC penetration rates compared to those with a higher AC penetration level, though the differences were statistically insignificant. Given the lack of individual-level AC usage data and a relatively smaller sample size for AC analysis compared to our main analysis (n = 60,487, 31.7%), future work is warranted for a deeper understanding of the role of individual AC use in protecting pregnant women. In addition, although AC use is an important intervention to avert health losses due to extreme heat, it has greenhouse gas and air pollution consequences due to its high electricity usage. Cool roofs with a higher albedo have been suggested as an alternative strategy to prevent mortality and possibly other adverse outcomes caused by heat (Macintyre and Heaviside, 2019).

We found stronger associations of heatwaves on PROM in younger mothers (< 25 years), with positive associations even for the most conservative heatwave definitions (i.e., HWD11 and HWD12), suggesting that younger mothers may be particularly vulnerable to extreme heat (> 98th percentile). Positive associations for the less intense heatwaves were also found for mothers aged 25–34 (HWD1-HWD4 and HWD7) and ≥ 35 (HWD1-HWD3 and HWD5). Our findings were partially consistent with a Chinese study in which stronger associations between extreme heat and the risk of PROM were shown in mothers younger than 35 years old (Song et al., 2019a). Although previous studies showed that Black mothers were at higher risks of PPROM (Goldenberg et al., 2008; Shen et al., 2008), we detected no statistically significant difference among race/ethnicity groups, and positive results were only found in Hispanic and non-Hispanic White populations in our study. Only 7% of our study population was Black, which may explain the imprecise results for this group. Besides, during the study period, the prevalence of all defined heatwaves in the African American population was lower than that in the Hispanic and non-Hispanic White populations. The possible reason might be that the African American population in our study was clustered in Los Angeles County (62.6%) where heatwaves are not as severe as those in inland areas of Southern California. Furthermore, mothers with lower education and income levels had significantly higher risks of heat-related PROM. Those mothers with lower socioeconomic status may live in a poor environment with less green space, have limited knowledge or resources to protect themselves from heat exposure, or suffer more from poor nutrition compared to well-educated mothers with higher incomes. Additionally, our study identified smoking as a positive effect modifier for heat effects on PROM. By inducing oxidative stress to accelerate fetal membrane apoptosis and proteolytic degradation, smoking can lead to the pathophysiological processes of PROM that may be further exacerbated by heat exposure (Ekwo et al., 1993; England et al., 2013), thus mothers who smoked would be more susceptible to heat-induced PROM. By identifying subpopulations with higher vulnerability to climate sensitive exposures, our results may have important implications for implementing targeted interventions or risk communication to help pregnant women reduce health losses in extremely hot weather.

Our study has several strengths. First, compared with previous publications, we had a larger sample size with a diverse population from the KPSC pregnancy cohort. Second, based on KPSC EHRs, we were able to obtain rich and high-quality clinical data, including spontaneous PROM outcome information, accurate PROM diagnosis dates, and residential histories over the entire pregnancy. Due to a lack of exact PROM dates, most previous studies on PROM used dates of delivery as the proxy (Ha et al., 2018; Song et al., 2019a; Wallace et al., 2016; Wang et al., 2019), which may lead to measurement errors, especially for acute exposure assessment. Third, we extensively investigated the effect modification by modifiable environmental factors, including air pollution, green space, and AC penetration.

Some limitations should be acknowledged. First, due to data unavailability, there was no consideration of maternal time-activity patterns or indoor/workplace exposure, which may lead to exposure misclassification. Without the individual-level AC exposure data, we used the census tract-level AC penetration rate in 2015 and 2016 as a proxy of AC accessibility for each subject. However, those data cannot reflect the short-term personal accessibility of AC during the exposure window of heatwaves in our study. Future studies may obtain the individual-level real-time AC usage data (e.g., the frequency and duration) during the same window of heat exposure to acquire more accurate results of effect modification. Second, since only monthly air pollution exposure data were obtained for this study, we did not examine the effect modification by more short-term exposure to air pollution, such as the exposure during the last gestational week. Similarly, by using the spatial snapshot data on residential green space derived from Bing Map street view images, we were unable to examine the sensitive time window of green space exposure as an effect modifier. A better understanding of effect modification by those modifiable environmental factors requires further studies using exposure data with a finer temporal resolution. Third, the study population mainly included residents in Southern California who may be more adapted to heatwaves than those living in cooler regions. Given that environmental factors could vary greatly across regions and countries, future studies in different climate zones are warranted (Sun et al., 2020a). In addition, as we used heat exposure data at a 4 km resolution, we did not examine local heat island effects that may be affected by surrounding buildings, pavement, proximity to roadways, etc., which deserve future exploration.

## 5. Conclusions

In this comprehensive evaluation of heatwave effects on spontaneous PROM as well as effect modification by air pollution, climate adaption factors, and maternal characteristics, we found elevated risks of PROM associated with acute heat exposure during the last gestational week in warm seasons. Targeted interventions might be beneficial especially for expecting mothers with higher air pollution exposure during the pregnancy, a younger age, lower socioeconomic status, and smoking behavior. Although effect modification by climate adaptation measures was not statistically significant, higher heat-related PROM risks were observed among mothers living with lower surrounding green space or lower AC penetration in this study.

## Supplementary Material

Supplemental Material

## Figures and Tables

**Table 1:** Descriptive statistics of participants and environmental factors during the warm season (May-September), 2008–2018.

Characteristics	Total subjects (n = 190,767)	Non PROM (n = 174,277)	PROM (n = 16,490)	TPROM (n = 12,515)	PPROM (n = 3,975)

Maternal age (n, %)					
< 25	36,915 (19.35)	33,709 (19.34)	3,206 (19.44)	2,444 (19.53)	762 (19.17)
25–34	113,685 (59.59)	103,785 (59.55)	9,900 (60.04)	7,610 (60.81)	2,290 (57.61)
≥ 35	40,167 (21.06)	36,783 (21.11)	3,384 (20.52)	2,461 (19.66)	923 (23.22)
Race/Ethnicity (n, %)					
African American	14,231 (7.46)	13,057 (7.49)	1,174 (7.12)	770 (6.15)	404 (10.16)
Asian	23,509 (12.32)	21,289 (12.22)	2,220 (13.46)	1,700 (13.58)	520 (13.08)
Hispanic	97,903 (51.33)	89,377 (51.29)	8,526 (51.71)	6,451 (51.55)	2,075 (52.20)
Non-Hispanic White	50,092 (26.26)	45,949 (26.37)	4,143 (25.13)	3,259 (26.04)	884 (22.24)
Multiple/ Others	5,011 (2.63)	4,585 (2.63)	426 (2.58)	334 (2.67)	92 (2.31)
Education level (n, %)					
< College	59,304 (31.69)	54,736 (32.02)	4,568 (28.25)	3,412 (27.77)	1,156 (29.79)
College	102,701 (54.89)	93,558 (54.73)	9,143 (56.55)	6,953 (56.58)	2,190 (56.43)
> College	25,104 (13.42)	22,646 (13.25)	2,458 (15.20)	1,923 (15.65)	535 (13.79)
BMI, kg/m^2^ (n, %)					
Underweight (<18.5)	4,647 (2.45)	4,231 (2.45)	416 (2.54)	313 (2.51)	103 (2.60)
Normal weight (18.5–24.9)	81,386 (42.98)	74,372 (43.00)	7,014 (42.76)	5,481 (44.03)	1,533 (38.74)
Overweight (25.0–29.9)	53,252 (28.12)	48,488 (28.04)	4,764 (29.04)	3,656 (29.37)	1,108 (28.00)
Obese (≥ 30.0)	50,061 (26.44)	45,851 (26.51)	4,210 (25.66)	2,997 (24.08)	1,213 (30.65)
Smoking status (n, %)					
Non-smoker	159,453 (83.59)	145,720 (83.62)	13,733 (83.29)	10,499 (83.89)	3,234 (81.38)
Ever smoker	21,614 (11.33)	19,723 (11.32)	1,891 (11.47)	1,413 (11.29)	478 (12.03)
Smoker during pregnancy	9,685 (5.08)	8,820 (5.06)	865 (5.25)	603 (4.82)	262 (6.59)
Parity (n, %)					
Primiparous	78,677 (41.30)	69,709 (40.05)	8,968 (54.44)	7,012 (56.06)	1,956 (49.32)
Multiparous	111,837 (58.70)	104,331 (59.95)	7,506 (45.56)	5,496 (43.94)	2,010 (50.68)
Income level, US Dollars (mean, SD)	59,730 (21,742)	59,720 (21,748)	59,838 (21,678)	60,036 (21,571)	59,211 (22,001)
Environmental factors (mean, SD)					
HI ^a^, °C	28.66 (3.90)	28.63 (3.90)	28.99 (3.89)	29.02 (3.89)	28.90 (3.89)
Daily max temp ^a^, °C	29.30 (4.78)	29.29 (4.78)	29.39 (4.82)	29.39 (4.79)	29.38 (4.91)
PM_2.5_^b^, μg/m^3^	11.40 (2.34)	11.43 (2.35)	11.20 (2.15)	11.20 (2.09)	11.17 (2.33)
PM_2.5_last 3 months_^c^, μg/ m^3^	11.35 (2.21)	11.35 (2.21)	11.26 (2.11)	11.27 (2.08)	11.23 (2.19)
PM_2.5_last month_^d^, μg/m^3^	11.76 (2.41)	11.76 (2.42)	11.77 (2.33)	11.79 (2.31)	11.71 (2.41)
NO_2_^b^, ppb	16.12 (4.10)	16.13 (4.11)	15.94 (3.98)	16.09 (3.85)	15.49 (4.31)
NO_2_last 3 months_^c^, ppb	12.03 (3.76)	12.04 (3.79)	11.94 (3.49)	11.95 (3.41)	11.93 (3.72)
NO_2_last month_^d^, ppb	11.74 (3.92)	11.76 (3.96)	11.58 (3.58)	11.58 (3.51)	11.58 (3.81)
Green space ^b^, %	25.33 (5.10)	25.32 (5.10)	25.47 (5.17)	25.51 (5.20)	25.31 (5.06)

Abbreviation: PROM, premature rupture of membranes; TPROM, term premature rupture of membranes; PPROM, preterm premature rupture of membranes;

BMI, body mass index; SD, standard deviation; HI, heat index; °C, degree Celsius; daily max temp, daily maximum temperature; ppb, parts per billion.

a The average exposure level in the last gestational week.

b The average exposure level throughout pregnancy.

c The average exposure level during the last three months of pregnancy.

d The average exposure level during the last month of pregnancy.

**Table 2:** Summary statistics of individual-experienced heatwaves in the last gestational week for the study population in the warm season (May-September), 2008–2018.

Heatwave	Definitions	Cut-off heat index values	Total Subjects experiencing heatwaves (n, %) ^a^	Total Subjects experiencing > 1 heatwaves (n, %) ^b^	Non-PROM experiencing heatwaves (n, %)	PROM experiencing heatwaves (n, %)

HWD1	Heat index > 75th percentile lasting for ≥ 2 days	32.8 °C	49,518 (25.96)	3,470 (1.82)	44,909 (25.77)	4,615 (27.99)
HWD2	Heat index > 75th percentile lasting for ≥ 3 days	32.8 °C	34,522 (18.10)	417 (0.22)	31,230 (17.92)	3,292 (19.96)
HWD3	Heat index > 75th percentile lasting for ≥ 4 days	32.8 °C	22,997 (12.06)	–	20,728 (11.89)	2,269 (13.76)
HWD4	Heat index > 90th percentile lasting for ≥ 2 days	35.6 °C	17,822 (9.34)	738 (0.39)	16,031 (9.20)	1,791 (10.86)
HWD5	Heat index > 90th percentile lasting for ≥ 3 days	35.6 °C	10,253 (5.37)	86 (0.05)	9,219 (5.29)	1,034 (6.27)
HWD6	Heat index > 90th percentile lasting for ≥ 4 days	35.6 °C	5,679 (2.98)	–	5,076 (2.91)	603 (3.66)
HWD7	Heat index > 95th percentile lasting for ≥ 2 days	37.8 °C	5,690 (2.98)	141 (0.07)	5,075 (2.91)	615 (3.73)
HWD8	Heat index > 95th percentile lasting for ≥ 3 days	37.8 °C	2,772 (1.45)	23 (0.01)	2,508 (1.44)	264 (1.60)
HWD9	Heat index > 95th percentile lasting for ≥ 4 days	37.8 °C	1,404 (0.74)	–	1,274 (0.73)	130 (0.79)
HWD10	Heat index > 98th percentile lasting for ≥ 2 days	40.0 °C	1,282 (0.67)	73 (0.04)	1,148 (0.66)	134 (0.81)
HWD11	Heat index > 98th percentile lasting for ≥ 3 days	40.0 °C	624 (0.33)	10 (0.01)	549 (0.32)	75 (0.45)
HWD12	Heat index > 98th percentile lasting for ≥ 4 days	40.0 °C	339 (0.18)	–	303 (0.17)	36 (0.22)

Abbreviation: PROM, premature rupture of membranes; HI, heat index; °C, degree Celsius.

a Number of mothers with and without PROM who experienced at least one heatwave event in the last week of pregnancy.

b Number of mothers with and without PROM who experienced two or more heatwave events in the last gestational week. Based on the definitions of HWD3, HWD6, HWD9, and HWD12, mothers will not experience more than one heatwave using these four definitions during the last gestational week.

## Data Availability

The data that has been used is confidential.
