# Supplementary material for: The role of extreme heat exposure on premature rupture of membranes in Southern California: A study from a large pregnancy cohort
Source: Environ Int. Author manuscript; Available in PMC 2024 Mar 6. (PMC10917632; doi:10.1016/j.envint.2023.107824)
Supplement: Supplemental Material [file NIHMS1967188-supplement-Supplemental_Material.docx]

**Supplementary Materials**

**The Role of Extreme Heat Exposure on Premature Rupture of Membranes in Southern California: A study from a Large Pregnancy Cohort**

Anqi Jiao^a^, Yi Sun^a^, David A. Sacks^b, c^, Chantal Avila^b^, Vicki Chiu^b^, John Molitor^d^, Jiu-Chiuan Chen^e^, Kelly T Sanders^f^, John T Abatzoglou^g^, Jeff Slezak^b^, Tarik Benmarhnia^h^, Darios Getahun^b, i *^, Jun Wu^a *^

^a^ Department of Environmental and Occupational Health, Program in Public Health, University of California, Irvine, CA, USA

^b^ Department of Research & Evaluation, Kaiser Permanente Southern California, Pasadena, CA, USA

^c^ Department of Obstetrics and Gynecology, University of Southern California, Keck School of Medicine, Los Angeles, CA

^d^ College of Public Health and Human Sciences, Oregon State University, Corvallis, OR 97331, USA

^e^ Department of Population and Public Health Sciences, University of Southern California, Los Angeles, CA 90033, USA

^f^ Department of Civil and Environmental Engineering, University of Southern California, CA, USA

^g^ School of Engineering, University of California, Merced, USA

^h^ Scripps Institution of Oceanography, University of California, San Diego, 9500 Gilman Drive #0725, CA La Jolla 92093, USA

^i^ Department of Health Systems Science, Kaiser Permanente Bernard J. Tyson School of Medicine, Pasadena, CA

* Co-Senior Authors

**Corresponding author**

Dr. Darios Getahun

100 S. Los Robles Avenue 2nd Floor

Pasadena, CA 91101, USA

Email: Darios.T.Getahun@kp.org

Tel: +1 626-564-5658

Dr. Jun Wu

856 Health Sciences Rd (Quad), Suite 3200

Irvine, CA 92697-1830, USA

Email: junwu@hs.uci.edu

Tel: +1 949-824-0548

**Table of Contents**

**Table S1.** Pearson correlation coefficients between environmental factors for the total study population.

**Table S2.** The estimated hazard ratios (HRs) with 95% confidence intervals (CIs) of PROM, TPROM, and PPROM associated with different heatwave definitions.

**Table S3.** Adjusted hazard ratios (HRs) and 95% confidence intervals (CIs) of PROM associated with heatwaves stratified by air pollution exposure during the entire pregnancy.

**Table S4.** Adjusted hazard ratios (HRs) and 95% confidence intervals (CIs) of PROM associated with heatwaves stratified by air pollution exposure during the last three months of pregnancy.

**Table S5.** Adjusted hazard ratios (HRs) and 95% confidence intervals (CIs) of PROM associated with heatwaves stratified by air pollution exposure during the last month of pregnancy.

**Figure S1.** The hazard ratios (HRs) of PROM associated with different heatwave definitions in groups with lower (< 50^th^) vs. higher (≥ 50^th^) exposure to PM_2.5_ (A) and NO_2_ (B) during the last three months of pregnancy.

**Figure S2.** The hazard ratios (HRs) of PROM associated with different heatwave definitions in groups with lower (< 50^th^) vs. higher (≥ 50^th^) exposure to PM_2.5_ (A) and NO_2_ (B) during the last month of pregnancy.

**Table S6.** Adjusted hazard ratios (HRs) and 95% confidence intervals (CIs) of PROM associated with heatwaves stratified by climate adaptation measures.

**Table S7.** Adjusted hazard ratios (HRs) and 95% confidence intervals (CIs) of PROM associated with heatwaves among population subgroups stratified by maternal characteristics.

**Table S8.** Adjusted hazard ratios (HRs) and 95% confidence intervals (CIs) of PROM associated with heatwaves of sensitivity analyses.

**Table S9.** The effect modification in associations between heatwaves and PROM by exposure to air pollution during the entire pregnancy estimated in models with the interaction term.

**Table S10.** The effect modification in associations between heatwaves and PROM by exposure to air pollution during the last three months of pregnancy estimated in models with the interaction term.

**Table S11.** The effect modification in associations between heatwaves and PROM by exposure to air pollution during the last month of pregnancy estimated in models with the interaction term.

**Table S12.** The effect modification in associations between heatwaves and PROM by climate adaptation measures estimated in models with the interaction term.

**Table S13.** The effect modification in associations between heatwaves and PROM by maternal characteristics estimated in models with the interaction term.

**Table S1.** Pearson correlation coefficients between environmental factors for the total study population.

|  | HI ^a^ | Daily max temp ^a^ | PM_2.5_ ^b^ | PM_2.5__  _last 3 months_ ^c^ | PM_2.5__  _last month_ ^d^ | NO_2_ ^b^ | NO_2__  _last 3 months_ ^c^ | NO_2__  _last month_ ^d^ |
| --- | --- | --- | --- | --- | --- | --- | --- | --- |
| Daily max temp ^a^ | 0.54** |  |  |  |  |  |  |  |
| PM_2.5_ ^b^ | -0.004 | -0.02** |  |  |  |  |  |  |
| PM_2.5_last 3 months_ ^c^ | 0.10** | 0.29** | 0.64** |  |  |  |  |  |
| PM_2.5_last month_ ^d^ | 0.17** | 0.30** | 0.55** | 0.89** |  |  |  |  |
| NO_2_ ^b^ | -0.07** | -0.21** | 0.62** | 0.38** | 0.38** |  |  |  |
| NO_2_last 3 months_ ^c^ | -0.10** | -0.05** | 0.52** | 0.46** | 0.39** | 0.81** |  |  |
| NO_2_last month_ ^d^ | -0.02** | 0.20** | 0.47** | 0.60** | 0.51** | 0.64** | 0.90** |  |
| Green space ^b^ | 0.01* | -0.01* | -0.06** | -0.002 | -0.003 | 0.01** | 0.02** | 0.02** |

***P* < 0.01, **P* < 0.05.

Abbreviation: HI, heat index; daily max temp, daily maximum temperature.

^a^ The average exposure level in the last gestational week.

^b^ The average exposure level throughout pregnancy.

^c^ The average exposure level during the last three months of pregnancy.

^d^ The average exposure level during the last month of pregnancy.

**Table S2.** The estimated hazard ratios (HRs) with 95% confidence intervals (CIs) of PROM, TPROM, and PPROM associated with different heatwave definitions.

| Heatwave definitions | Models | | | |
| --- | --- | --- | --- | --- |
|  | Unadjusted-PROM | Adjusted-  PROM ^a^ | Adjusted-  TPROM ^a^ | Adjusted-  PPROM ^a^ |
| HWD1 | **1.097 (1.058, 1.138)** | **1.087 (1.049, 1.127)** | **1.070 (1.027, 1.115)** | **1.133 (1.056, 1.215)** |
| HWD2 | **1.125 (1.081, 1.172)** | **1.106 (1.062, 1.151)** | **1.097 (1.047, 1.148)** | **1.126 (1.041, 1.219)** |
| HWD3 | **1.160 (1.107, 1.215)** | **1.124 (1.073, 1.177)** | **1.121 (1.063, 1.182)** | **1.125 (1.025, 1.234)** |
| HWD4 | **1.180 (1.121, 1.242)** | **1.122 (1.067, 1.181)** | **1.121 (1.057, 1.187)** | **1.118 (1.008, 1.241)** |
| HWD5 | **1.193 (1.118, 1.274)** | **1.125 (1.055, 1.201)** | **1.131 (1.050, 1.218)** | 1.113 (0.974, 1.271) |
| HWD6 | **1.260 (1.158, 1.370)** | **1.142 (1.050, 1.241)** | **1.163 (1.058, 1.277)** | 1.079 (0.903, 1.291) |
| HWD7 | **1.270 (1.169, 1.380)** | **1.137 (1.047, 1.235)** | **1.142 (1.041, 1.254)** | 1.119 (0.939, 1.333) |
| HWD8 | 1.104 (0.974, 1.252) | 0.996 (0.879, 1.128) | 0.985 (0.855, 1.135) | 1.059 (0.819, 1.368) |
| HWD9 | 1.008 (0.904, 1.291) | 0.995 (0.835, 1.187) | 0.936 (0.762, 1.150) | 1.233 (0.882, 1.724) |
| HWD10 | **1.223 (1.025, 1.458)** | 0.999 (0.839, 1.189) | 0.995 (0.819, 1.209) | 1.033 (0.706, 1.511) |
| HWD11 | **1.456 (1.150, 1.843)** | 1.250 (0.990, 1.577) | 1.189 (0.908, 1.557) | 1.527 (0.971, 2.403) |
| HWD12 | 1.286 (0.918, 1.801) | 1.127 (0.808, 1.573) | 1.096 (0.746, 1.611) | 1.354 (0.702, 2.610) |

Note: Bold font face indicates a statistically significant result (*P* < 0.05).

^a^ Models are adjusted for maternal age, race/ethnicity, education level, median family household income, pre-pregnancy BMI, smoking status, year of infant birth, and parity.

**Table S3.** Adjusted hazard ratios (HRs) and 95% confidence intervals (CIs) of PROM associated with heatwaves stratified by air pollution exposure during the entire pregnancy.

| Description | | HRs | 95% CI | | *P* value for Cochran's Q test |
| --- | --- | --- | --- | --- | --- |
| PM_2.5_ | | | | | |
| HWD1 | < 50^th^ | **1.074** | **1.020** | **1.130** | 0.172 |
|  | ≥ 50^th^ | **1.131** | **1.070** | **1.195** |  |
| HWD2 | < 50^th^ | **1.103** | **1.042** | **1.168** | 0.451 |
|  | ≥ 50^th^ | **1.139** | **1.070** | **1.211** |  |
| HWD3 | < 50^th^ | **1.115** | **1.044** | **1.191** | 0.523 |
|  | ≥ 50^th^ | **1.151** | **1.071** | **1.237** |  |
| HWD4 | < 50^th^ | **1.114** | **1.038** | **1.195** | 0.264 |
|  | ≥ 50^th^ | **1.185** | **1.091** | **1.287** |  |
| HWD5 | < 50^th^ | **1.124** | **1.028** | **1.230** | 0.565 |
|  | ≥ 50^th^ | **1.169** | **1.053** | **1.299** |  |
| HWD6 | < 50^th^ | 1.102 | 0.984 | 1.234 | 0.136 |
|  | ≥ 50^th^ | **1.262** | **1.099** | **1.449** |  |
| HWD7 | < 50^th^ | 1.080 | 0.961 | 1.213 | 0.097 |
|  | ≥ 50^th^ | **1.254** | **1.098** | **1.432** |  |
| HWD8 | < 50^th^ | 0.936 | 0.788 | 1.111 | 0.325 |
|  | ≥ 50^th^ | 1.072 | 0.869 | 1.322 |  |
| HWD9 | < 50^th^ | 0.881 | 0.697 | 1.115 | 0.087 |
|  | ≥ 50^th^ | 1.239 | 0.908 | 1.691 |  |
| HWD10 | < 50^th^ | 0.957 | 0.762 | 1.202 | 0.510 |
|  | ≥ 50^th^ | 1.092 | 0.790 | 1.511 |  |
| HWD11 | < 50^th^ | 1.149 | 0.854 | 1.547 | 0.288 |
|  | ≥ 50^th^ | 1.557 | 0.968 | 2.504 |  |
| HWD12 | < 50^th^ | 1.136 | 0.759 | 1.700 | 0.925 |
|  | ≥ 50^th^ | 1.152 | 0.543 | 2.443 |  |
| NO_2_ | | | | | |
| HWD1 | < 50^th^ | **1.128** | **1.071** | **1.187** | 0.102 |
|  | ≥ 50^th^ | **1.059** | **1.002** | **1.120** |  |
| HWD2 | < 50^th^ | **1.143** | **1.081** | **1.209** | 0.240 |
|  | ≥ 50^th^ | **1.086** | **1.019** | **1.158** |  |
| HWD3 | < 50^th^ | **1.141** | **1.070** | **1.217** | 0.659 |
|  | ≥ 50^th^ | **1.116** | **1.035** | **1.202** |  |
| HWD4 | < 50^th^ | **1.138** | **1.062** | **1.220** | 0.891 |
|  | ≥ 50^th^ | **1.146** | **1.054** | **1.247** |  |
| HWD5 | < 50^th^ | **1.163** | **1.067** | **1.267** | 0.629 |
|  | ≥ 50^th^ | **1.123** | **1.003** | **1.258** |  |
| HWD6 | < 50^th^ | **1.129** | **1.014** | **1.256** | 0.252 |
|  | ≥ 50^th^ | **1.258** | **1.080** | **1.464** |  |
| HWD7 | < 50^th^ | **1.121** | **1.004** | **1.251** | 0.561 |
|  | ≥ 50^th^ | **1.179** | **1.021** | **1.361** |  |
| HWD8 | < 50^th^ | 0.958 | 0.818 | 1.121 | 0.545 |
|  | ≥ 50^th^ | 1.045 | 0.817 | 1.335 |  |
| HWD9 | < 50^th^ | 0.937 | 0.760 | 1.156 | 0.390 |
|  | ≥ 50^th^ | 1.143 | 0.755 | 1.730 |  |
| HWD10 | < 50^th^ | 0.957 | 0.771 | 1.187 | 0.464 |
|  | ≥ 50^th^ | 1.119 | 0.774 | 1.616 |  |
| HWD11 | < 50^th^ | 1.192 | 0.911 | 1.561 | 0.454 |
|  | ≥ 50^th^ | 1.583 | 0.787 | 3.184 |  |
| HWD12 | < 50^th^ | 1.145 | 0.793 | 1.653 | 0.928 |
|  | ≥ 50^th^ | 1.103 | 0.272 | 4.476 |  |

Note: Models are adjusted for maternal age, race/ethnicity, education level, median family household income, pre-pregnancy BMI, smoking status, year of infant birth, and parity. The cutoff concentrations of PM_2.5_ and NO_2_ are 11.26 µg/m^3^ and 16.45 ppb, respectively. Bold font face indicates a statistically significant result (*P* < 0.05). The *P* value refers to the comparison between subgroups and is obtained from Cochran’s Q test.

**Table S4.** Adjusted hazard ratios (HRs) and 95% confidence intervals (CIs) of PROM associated with heatwaves stratified by air pollution exposure during the last three months of pregnancy.

| Description | | HRs | 95% CI | | *P* value for Cochran's Q test |
| --- | --- | --- | --- | --- | --- |
| PM_2.5_ | | | | | |
| HWD1 | < 50^th^ | 1.049 | 0.995 | 1.107 | **0.021** |
|  | ≥ 50^th^ | **1.141** | **1.085** | **1.199** |  |
| HWD2 | < 50^th^ | 1.054 | 0.992 | 1.119 | **0.009** |
|  | ≥ 50^th^ | **1.173** | **1.109** | **1.240** |  |
| HWD3 | < 50^th^ | **1.102** | **1.029** | **1.180** | 0.349 |
|  | ≥ 50^th^ | **1.152** | **1.080** | **1.229** |  |
| HWD4 | < 50^th^ | **1.096** | **1.018** | **1.180** | 0.215 |
|  | ≥ 50^th^ | **1.168** | **1.087** | **1.257** |  |
| HWD5 | < 50^th^ | **1.140** | **1.038** | **1.253** | 0.654 |
|  | ≥ 50^th^ | **1.144** | **1.043** | **1.255** |  |
| HWD6 | < 50^th^ | **1.154** | **1.025** | **1.300** | 0.596 |
|  | ≥ 50^th^ | **1.178** | **1.044** | **1.330** |  |
| HWD7 | < 50^th^ | **1.162** | **1.034** | **1.305** | 0.708 |
|  | ≥ 50^th^ | **1.132** | **1.003** | **1.278** |  |
| HWD8 | < 50^th^ | 1.040 | 0.871 | 1.242 | 0.516 |
|  | ≥ 50^th^ | 0.956 | 0.796 | 1.147 |  |
| HWD9 | < 50^th^ | 1.047 | 0.814 | 1.347 | 0.568 |
|  | ≥ 50^th^ | 0.943 | 0.730 | 1.218 |  |
| HWD10 | < 50^th^ | 1.007 | 0.802 | 1.265 | 0.945 |
|  | ≥ 50^th^ | 0.999 | 0.753 | 1.325 |  |
| HWD11 | < 50^th^ | 1.261 | 0.923 | 1.723 | 0.780 |
|  | ≥ 50^th^ | 1.187 | 0.820 | 1.718 |  |
| HWD12 | < 50^th^ | 1.239 | 0.828 | 1.854 | 0.508 |
|  | ≥ 50^th^ | 0.968 | 0.517 | 1.811 |  |
| NO_2_ | | | | | |
| HWD1 | < 50^th^ | **1.099** | **1.044** | **1.158** | 0.406 |
|  | ≥ 50^th^ | **1.108** | **1.053** | **1.167** |  |
| HWD2 | < 50^th^ | **1.112** | **1.051** | **1.177** | 0.357 |
|  | ≥ 50^th^ | **1.136** | **1.071** | **1.204** |  |
| HWD3 | < 50^th^ | **1.117** | **1.047** | **1.191** | 0.335 |
|  | ≥ 50^th^ | **1.162** | **1.085** | **1.245** |  |
| HWD4 | < 50^th^ | **1.127** | **1.050** | **1.210** | 0.529 |
|  | ≥ 50^th^ | **1.149** | **1.065** | **1.239** |  |
| HWD5 | < 50^th^ | **1.174** | **1.077** | **1.281** | 0.335 |
|  | ≥ 50^th^ | **1.131** | **1.022** | **1.252** |  |
| HWD6 | < 50^th^ | **1.169** | **1.049** | **1.303** | 0.483 |
|  | ≥ 50^th^ | **1.187** | **1.037** | **1.359** |  |
| HWD7 | < 50^th^ | **1.194** | **1.070** | **1.333** | 0.381 |
|  | ≥ 50^th^ | 1.130 | 0.992 | 1.288 |  |
| HWD8 | < 50^th^ | 0.998 | 0.849 | 1.173 | 0.646 |
|  | ≥ 50^th^ | 1.045 | 0.851 | 1.283 |  |
| HWD9 | < 50^th^ | 0.963 | 0.771 | 1.202 | 0.399 |
|  | ≥ 50^th^ | 1.125 | 0.830 | 1.524 |  |
| HWD10 | < 50^th^ | 0.982 | 0.781 | 1.236 | 0.509 |
|  | ≥ 50^th^ | 1.095 | 0.829 | 1.448 |  |
| HWD11 | < 50^th^ | 1.278 | 0.957 | 1.707 | 0.871 |
|  | ≥ 50^th^ | 1.233 | 0.807 | 1.885 |  |
| HWD12 | < 50^th^ | 1.219 | 0.833 | 1.784 | 0.637 |
|  | ≥ 50^th^ | 1.026 | 0.484 | 2.172 |  |

Note: Models are adjusted for maternal age, race/ethnicity, education level, median family household income, pre-pregnancy BMI, smoking status, year of infant birth, and parity. The cutoff concentrations of PM_2.5_ and NO_2_ are 10.98 µg/m^3^ and 11.76 ppb, respectively. Bold font face indicates a statistically significant result (*P* < 0.05). The *P* value refers to the comparison between subgroups and is obtained from Cochran’s Q test.

**Table S5.** Adjusted hazard ratios (HRs) and 95% confidence intervals (CIs) of PROM associated with heatwaves stratified by air pollution exposure during the last month of pregnancy.

| Description | | HRs | 95% CI | | *P* value for Cochran's Q test |
| --- | --- | --- | --- | --- | --- |
| PM_2.5_ | | | | | |
| HWD1 | < 50^th^ | 1.051 | 0.994 | 1.112 | 0.133 |
|  | ≥ 50^th^ | **1.112** | **1.060** | **1.166** |  |
| HWD2 | < 50^th^ | **1.075** | **1.009** | **1.146** | 0.273 |
|  | ≥ 50^th^ | **1.126** | **1.069** | **1.187** |  |
| HWD3 | < 50^th^ | **1.134** | **1.052** | **1.222** | 0.585 |
|  | ≥ 50^th^ | **1.107** | **1.043** | **1.176** |  |
| HWD4 | < 50^th^ | **1.101** | **1.017** | **1.193** | 0.393 |
|  | ≥ 50^th^ | **1.150** | **1.075** | **1.230** |  |
| HWD5 | < 50^th^ | **1.160** | **1.047** | **1.287** | 0.563 |
|  | ≥ 50^th^ | **1.126** | **1.034** | **1.226** |  |
| HWD6 | < 50^th^ | **1.175** | **1.031** | **1.339** | 0.662 |
|  | ≥ 50^th^ | **1.148** | **1.028** | **1.281** |  |
| HWD7 | < 50^th^ | **1.204** | **1.062** | **1.366** | 0.328 |
|  | ≥ 50^th^ | 1.111 | 0.994 | 1.242 |  |
| HWD8 | < 50^th^ | 1.010 | 0.826 | 1.235 | 0.890 |
|  | ≥ 50^th^ | 0.994 | 0.845 | 1.169 |  |
| HWD9 | < 50^th^ | 1.113 | 0.853 | 1.454 | 0.219 |
|  | ≥ 50^th^ | 0.889 | 0.697 | 1.132 |  |
| HWD10 | < 50^th^ | 1.103 | 0.863 | 1.410 | 0.303 |
|  | ≥ 50^th^ | 0.917 | 0.712 | 1.182 |  |
| HWD11 | < 50^th^ | 1.360 | 0.979 | 1.888 | 0.506 |
|  | ≥ 50^th^ | 1.159 | 0.824 | 1.630 |  |
| HWD12 | < 50^th^ | 1.305 | 0.865 | 1.970 | 0.394 |
|  | ≥ 50^th^ | 0.971 | 0.535 | 1.761 |  |
| NO_2_ | | | | | |
| HWD1 | < 50^th^ | **1.085** | **1.030** | **1.143** | 0.379 |
|  | ≥ 50^th^ | **1.112** | **1.058** | **1.170** |  |
| HWD2 | < 50^th^ | **1.085** | **1.024** | **1.150** | 0.107 |
|  | ≥ 50^th^ | **1.154** | **1.090** | **1.221** |  |
| HWD3 | < 50^th^ | **1.105** | **1.035** | **1.181** | 0.197 |
|  | ≥ 50^th^ | **1.170** | **1.095** | **1.250** |  |
| HWD4 | < 50^th^ | **1.128** | **1.050** | **1.211** | 0.449 |
|  | ≥ 50^th^ | **1.154** | **1.071** | **1.243** |  |
| HWD5 | < 50^th^ | **1.150** | **1.052** | **1.258** | 0.397 |
|  | ≥ 50^th^ | **1.164** | **1.057** | **1.281** |  |
| HWD6 | < 50^th^ | **1.162** | **1.039** | **1.301** | 0.434 |
|  | ≥ 50^th^ | **1.197** | **1.054** | **1.360** |  |
| HWD7 | < 50^th^ | **1.172** | **1.048** | **1.310** | 0.565 |
|  | ≥ 50^th^ | **1.153** | **1.016** | **1.309** |  |
| HWD8 | < 50^th^ | 0.988 | 0.835 | 1.168 | 0.468 |
|  | ≥ 50^th^ | 1.069 | 0.882 | 1.296 |  |
| HWD9 | < 50^th^ | 0.966 | 0.771 | 1.209 | 0.516 |
|  | ≥ 50^th^ | 1.089 | 0.810 | 1.466 |  |
| HWD10 | < 50^th^ | 1.007 | 0.804 | 1.261 | 0.636 |
|  | ≥ 50^th^ | 1.069 | 0.804 | 1.420 |  |
| HWD11 | < 50^th^ | 1.329 | 0.998 | 1.770 | 0.578 |
|  | ≥ 50^th^ | 1.155 | 0.756 | 1.765 |  |
| HWD12 | < 50^th^ | 1.237 | 0.845 | 1.811 | 0.519 |
|  | ≥ 50^th^ | 0.955 | 0.451 | 2.021 |  |

Note: Models are adjusted for maternal age, race/ethnicity, education level, median family household income, pre-pregnancy BMI, smoking status, year of infant birth, and parity. The cutoff concentrations of PM_2.5_ and NO_2_ are 11.39 µg/m^3^ and 11.37 ppb, respectively. Bold font face indicates a statistically significant result (*P* < 0.05). The *P* value refers to the comparison between subgroups and is obtained from Cochran’s Q test.


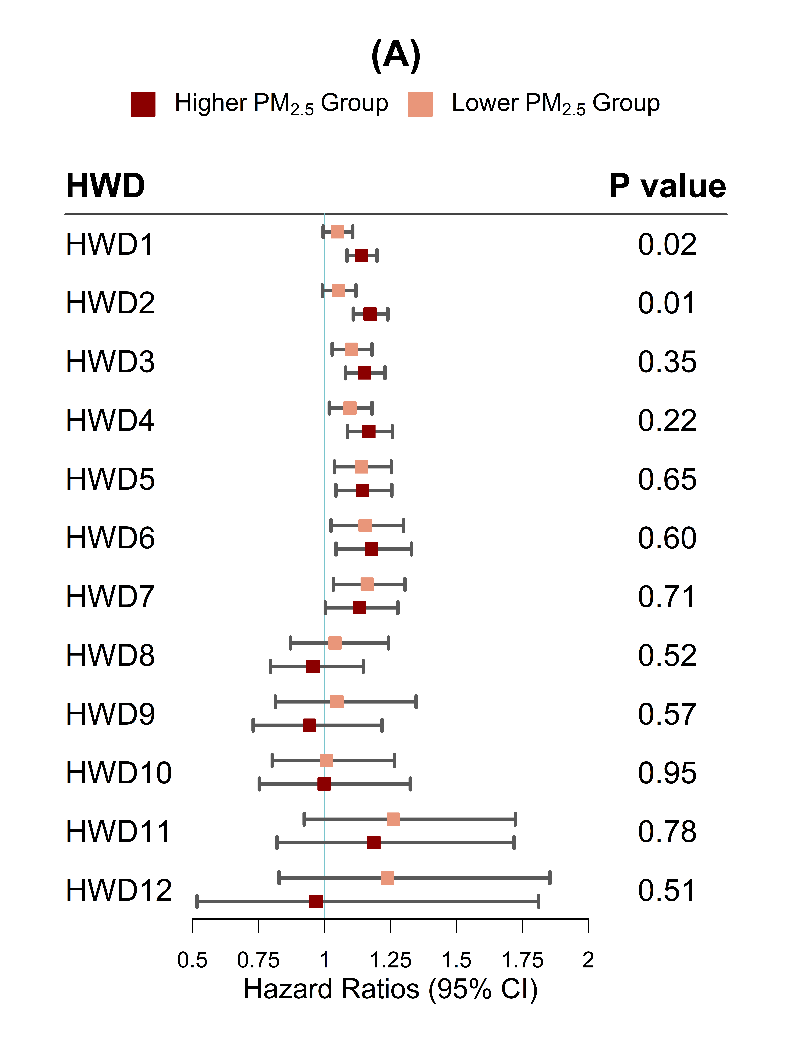

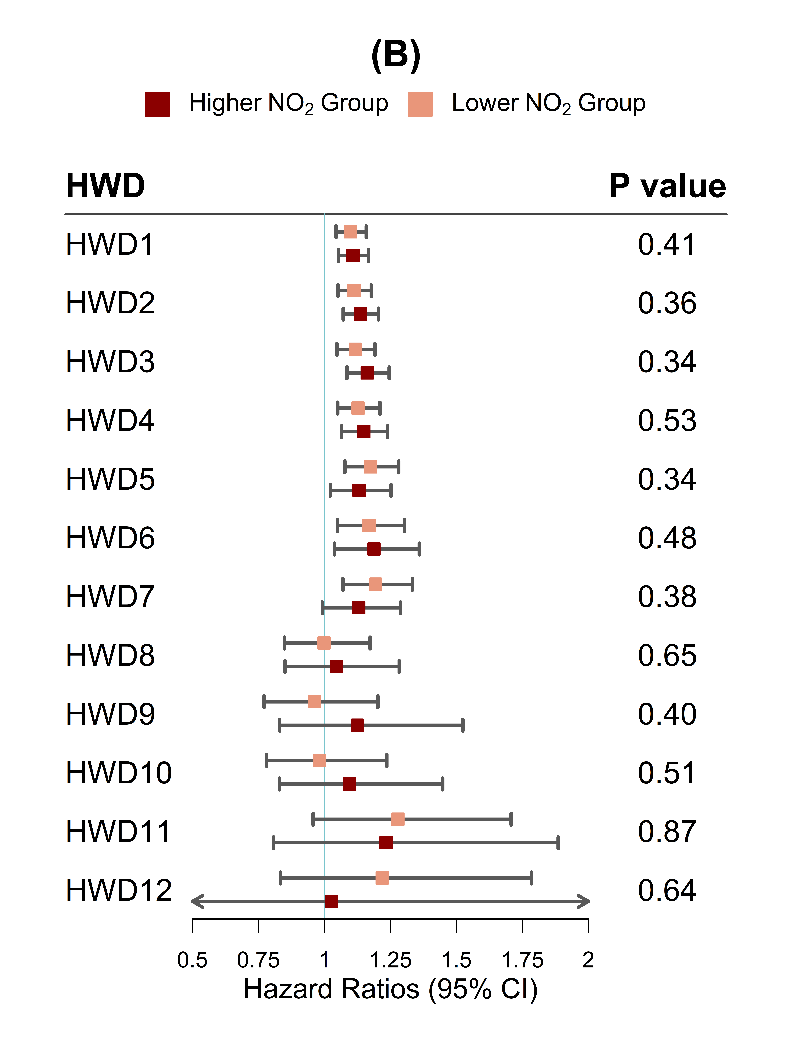


**Figure S1.** The hazard ratios (HRs) of PROM associated with different heatwave definitions in groups with lower (< 50^th^) vs. higher (≥ 50^th^) exposure to PM_2.5_ (A) and NO_2_ (B) during the last three months of pregnancy. The cutoff concentrations of PM_2.5_ and NO_2_ are 10.98 µg/m^3^ and 11.76 ppb, respectively. Models are adjusted for maternal age, race/ethnicity, education level, median family household income, pre-pregnancy BMI, smoking status, year of infant birth, and parity. The *P* value refers to the comparison between subgroups and is obtained from Cochran’s Q test.


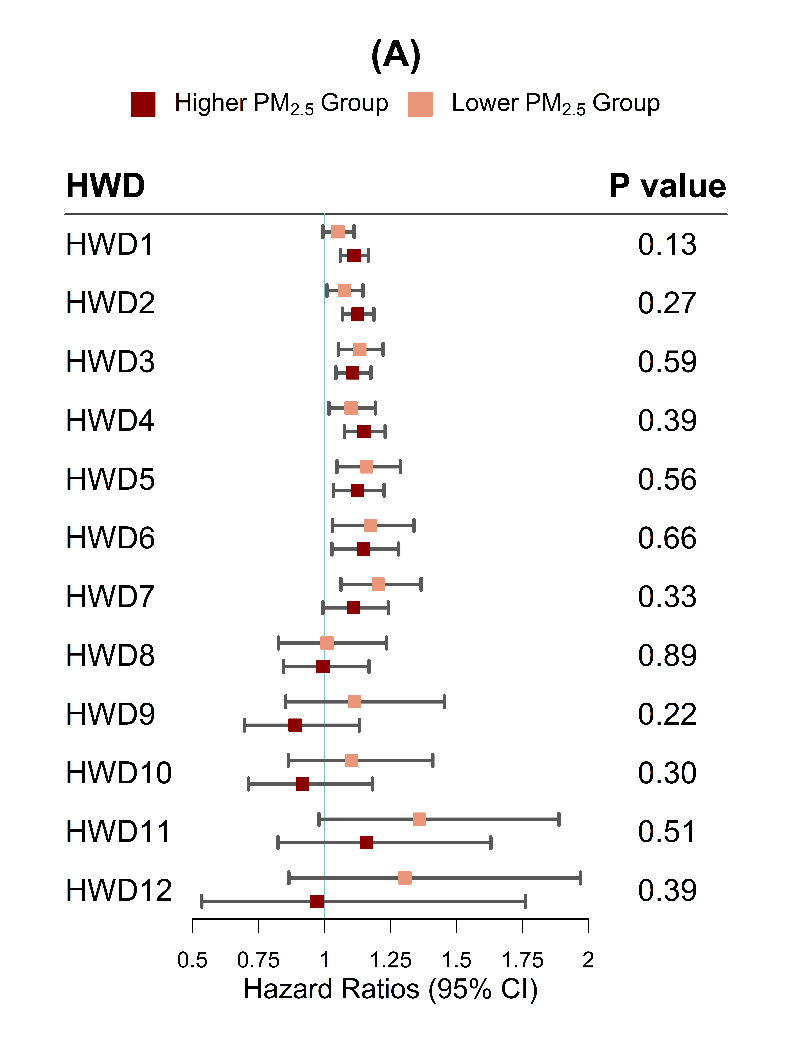

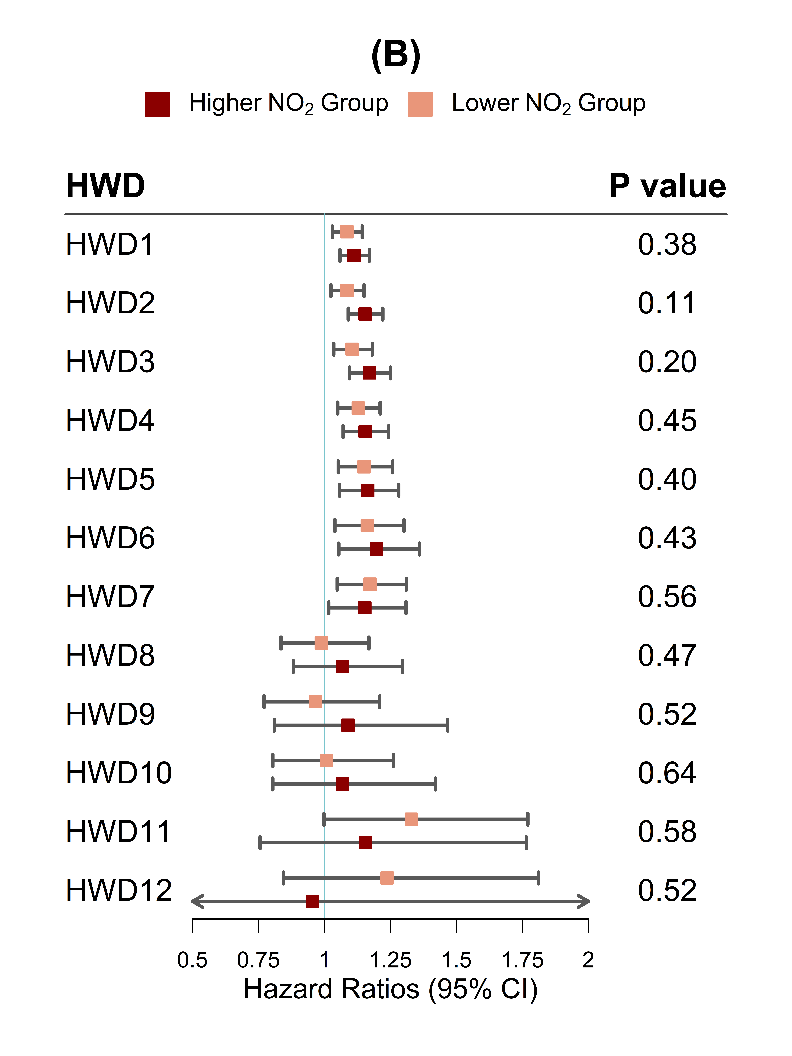


**Figure S2.** The hazard ratios (HRs) of PROM associated with different heatwave definitions in groups with lower (< 50^th^) vs. higher (≥ 50^th^) exposure to PM_2.5_ (A) and NO_2_ (B) during the last month of pregnancy. The cutoff concentrations of PM_2.5_ and NO_2_ are 11.39 µg/m^3^ and 11.37 ppb, respectively. Models are adjusted for maternal age, race/ethnicity, education level, median family household income, pre-pregnancy BMI, smoking status, year of infant birth, and parity. The *P* value refers to the comparison between subgroups and is obtained from Cochran’s Q test.

**Table S6.** Adjusted hazard ratios (HRs) and 95% confidence intervals (CIs) of PROM associated with heatwaves stratified by climate adaptation measures.

| Description | | HRs | 95% CI | | *P* value for Cochran's Q test |
| --- | --- | --- | --- | --- | --- |
| Green space | | | | | |
| HWD1 | < 50^th^ | **1.108** | **1.053** | **1.166** | 0.475 |
|  | ≥ 50^th^ | **1.084** | **1.031** | **1.139** |  |
| HWD2 | < 50^th^ | **1.134** | **1.072** | **1.200** | 0.330 |
|  | ≥ 50^th^ | **1.092** | **1.032** | **1.155** |  |
| HWD3 | < 50^th^ | **1.162** | **1.090** | **1.239** | 0.196 |
|  | ≥ 50^th^ | **1.094** | **1.024** | **1.169** |  |
| HWD4 | < 50^th^ | **1.172** | **1.092** | **1.257** | 0.102 |
|  | ≥ 50^th^ | **1.077** | **1.001** | **1.160** |  |
| HWD5 | < 50^th^ | **1.194** | **1.094** | **1.304** | 0.081 |
|  | ≥ 50^th^ | 1.063 | 0.966 | 1.171 |  |
| HWD6 | < 50^th^ | **1.224** | **1.097** | **1.366** | 0.104 |
|  | ≥ 50^th^ | 1.064 | 0.935 | 1.210 |  |
| HWD7 | < 50^th^ | **1.231** | **1.103** | **1.373** | 0.080 |
|  | ≥ 50^th^ | 1.062 | 0.937 | 1.204 |  |
| HWD8 | < 50^th^ | 1.098 | 0.939 | 1.284 | 0.110 |
|  | ≥ 50^th^ | 0.892 | 0.727 | 1.095 |  |
| HWD9 | < 50^th^ | 1.115 | 0.902 | 1.378 | 0.146 |
|  | ≥ 50^th^ | 0.842 | 0.614 | 1.157 |  |
| HWD10 | < 50^th^ | 1.120 | 0.906 | 1.383 | 0.164 |
|  | ≥ 50^th^ | 0.864 | 0.636 | 1.173 |  |
| HWD11 | < 50^th^ | **1.405** | **1.066** | **1.852** | 0.268 |
|  | ≥ 50^th^ | 1.057 | 0.686 | 1.627 |  |
| HWD12 | < 50^th^ | 1.221 | 0.836 | 1.783 | 0.613 |
|  | ≥ 50^th^ | 1.003 | 0.500 | 2.012 |  |
| AC penetration rate | | | | | |
| HWD1 | < 50^th^ | **1.083** | **1.002** | **1.171** | 0.355 |
|  | ≥ 50^th^ | 1.029 | 0.954 | 1.110 |  |
| HWD2 | < 50^th^ | **1.125** | **1.034** | **1.224** | 0.307 |
|  | ≥ 50^th^ | 1.060 | 0.979 | 1.148 |  |
| HWD3 | < 50^th^ | **1.145** | **1.041** | **1.258** | 0.336 |
|  | ≥ 50^th^ | 1.076 | 0.985 | 1.175 |  |
| HWD4 | < 50^th^ | **1.168** | **1.052** | **1.297** | 0.236 |
|  | ≥ 50^th^ | 1.071 | 0.971 | 1.181 |  |
| HWD5 | < 50^th^ | **1.148** | **1.006** | **1.309** | 0.534 |
|  | ≥ 50^th^ | 1.085 | 0.961 | 1.226 |  |
| HWD6 | < 50^th^ | 1.089 | 0.914 | 1.298 | 0.521 |
|  | ≥ 50^th^ | **1.173** | **1.009** | **1.363** |  |
| HWD7 | < 50^th^ | 1.148 | 0.970 | 1.360 | 0.690 |
|  | ≥ 50^th^ | 1.096 | 0.941 | 1.278 |  |
| HWD8 | < 50^th^ | 1.097 | 0.851 | 1.414 | 0.557 |
|  | ≥ 50^th^ | 0.992 | 0.796 | 1.236 |  |
| HWD9 | < 50^th^ | 1.231 | 0.867 | 1.747 | 0.313 |
|  | ≥ 50^th^ | 0.968 | 0.709 | 1.320 |  |
| HWD10 | < 50^th^ | 1.304 | 0.905 | 1.880 | 0.191 |
|  | ≥ 50^th^ | 0.948 | 0.691 | 1.303 |  |
| HWD11 | < 50^th^ | 1.563 | 0.946 | 2.584 | 0.417 |
|  | ≥ 50^th^ | 1.183 | 0.747 | 1.875 |  |
| HWD12 | < 50^th^ | 1.551 | 0.728 | 3.303 | 0.333 |
|  | ≥ 50^th^ | 0.948 | 0.489 | 1.839 |  |

Note: Models are adjusted for maternal age, race/ethnicity, education level, median family household income, pre-pregnancy BMI, smoking status, year of infant birth, and parity. The cutoff points of subgroups are 24.24% and 82% for green space and AC penetration rates, respectively. Bold font face indicates a statistically significant result (*P* < 0.05). The *P* value refers to the comparison between subgroups and is obtained from Cochran’s Q test.

**Table S7.** Adjusted hazard ratios (HRs) and 95% confidence intervals (CIs) of PROM associated with heatwaves among population subgroups stratified by maternal characteristics.

| Description | | HRs | 95% CI | | *P* value for Cochran's Q test |
| --- | --- | --- | --- | --- | --- |
| Maternal Age | | | | | |
| HWD1 | < 25 | **1.172** | **1.086** | **1.265** | 0.150 |
|  | 25-34 | **1.081** | **1.033** | **1.132** |  |
|  | ≥ 35 | **1.084** | **1.001** | **1.173** |  |
| HWD2 | < 25 | **1.230** | **1.132** | **1.337** | **0.032** |
|  | 25-34 | **1.083** | **1.029** | **1.140** |  |
|  | ≥ 35 | **1.114** | **1.019** | **1.219** |  |
| HWD3 | < 25 | **1.239** | **1.128** | **1.361** | 0.110 |
|  | 25-34 | **1.107** | **1.043** | **1.175** |  |
|  | ≥ 35 | **1.115** | **1.002** | **1.241** |  |
| HWD4 | < 25 | **1.264** | **1.140** | **1.401** | 0.064 |
|  | 25-34 | **1.102** | **1.032** | **1.177** |  |
|  | ≥ 35 | 1.104 | 0.981 | 1.242 |  |
| HWD5 | < 25 | **1.294** | **1.138** | **1.472** | 0.060 |
|  | 25-34 | 1.080 | 0.991 | 1.176 |  |
|  | ≥ 35 | **1.164** | **1.003** | **1.350** |  |
| HWD6 | < 25 | **1.360** | **1.160** | **1.595** | 0.063 |
|  | 25-34 | 1.085 | 0.971 | 1.211 |  |
|  | ≥ 35 | 1.111 | 0.906 | 1.362 |  |
| HWD7 | < 25 | **1.320** | **1.122** | **1.554** | 0.172 |
|  | 25-34 | **1.115** | **1.001** | **1.242** |  |
|  | ≥ 35 | 1.090 | 0.895 | 1.327 |  |
| HWD8 | < 25 | 1.225 | 0.977 | 1.537 | 0.155 |
|  | 25-34 | 0.944 | 0.800 | 1.114 |  |
|  | ≥ 35 | 0.939 | 0.681 | 1.296 |  |
| HWD9 | < 25 | **1.354** | **1.001** | **1.831** | 0.058 |
|  | 25-34 | 0.932 | 0.736 | 1.181 |  |
|  | ≥ 35 | 0.733 | 0.440 | 1.220 |  |
| HWD10 | < 25 | 1.267 | 0.930 | 1.728 | 0.109 |
|  | 25-34 | 1.006 | 0.803 | 1.260 |  |
|  | ≥ 35 | 0.662 | 0.384 | 1.144 |  |
| HWD11 | < 25 | **1.899** | **1.292** | **2.793** | **0.036** |
|  | 25-34 | 1.143 | 0.836 | 1.562 |  |
|  | ≥ 35 | 0.750 | 0.356 | 1.580 |  |
| HWD12 | < 25 | **2.004** | **1.236** | **3.248** | **0.017** |
|  | 25-34 | 1.007 | 0.638 | 1.587 |  |
|  | ≥ 35^a^ | - | - | - |  |
| Maternal race/ethnicity | | | | | |
| HWD1 | African American | 1.064 | 0.926 | 1.222 | 0.818 |
|  | Asian | 1.077 | 0.975 | 1.190 |  |
|  | Hispanic | **1.101** | **1.049** | **1.156** |  |
|  | Non-Hispanic White | **1.116** | **1.040** | **1.197** |  |
| HWD2 | African American | 1.031 | 0.881 | 1.208 | 0.635 |
|  | Asian | 1.117 | 0.996 | 1.252 |  |
|  | Hispanic | **1.120** | **1.062** | **1.182** |  |
|  | Non-Hispanic White | **1.144** | **1.059** | **1.237** |  |
| HWD3 | African American | 1.022 | 0.850 | 1.229 | 0.685 |
|  | Asian | **1.150** | **1.003** | **1.319** |  |
|  | Hispanic | **1.139** | **1.070** | **1.211** |  |
|  | Non-Hispanic White | **1.146** | **1.048** | **1.253** |  |

Note: Bold font face indicates a statistically significant result (*P* < 0.05). The P value refers to the comparison among subgroups and is obtained from Cochran’s Q test.

^a^ No PROM case more than 35 years old experienced HWD12 in this study.

**Table S7.** Adjusted hazard ratios (HRs) and 95% confidence intervals (CIs) of PROM associated with heatwaves among population subgroups stratified by maternal characteristics (continued).

| Description | | HRs | 95% CI | | *P* value for Cochran's Q test |
| --- | --- | --- | --- | --- | --- |
| Maternal race/ethnicity | | | | | |
| HWD4 | African American | 0.887 | 0.714 | 1.104 | 0.115 |
|  | Asian | 1.119 | 0.962 | 1.301 |  |
|  | Hispanic | **1.168** | **1.092** | **1.250** |  |
|  | Non-Hispanic White | **1.144** | **1.037** | **1.263** |  |
| HWD5 | African American | 1.010 | 0.764 | 1.335 | 0.713 |
|  | Asian | 1.105 | 0.906 | 1.348 |  |
|  | Hispanic | **1.169** | **1.074** | **1.274** |  |
|  | Non-Hispanic White | 1.127 | 0.993 | 1.280 |  |
| HWD6 | African American | 1.165 | 0.832 | 1.631 | 0.975 |
|  | Asian | 1.096 | 0.836 | 1.436 |  |
|  | Hispanic | **1.164** | **1.042** | **1.300** |  |
|  | Non-Hispanic White | 1.144 | 0.974 | 1.345 |  |
| HWD7 | African American | 1.189 | 0.842 | 1.680 | 0.084 |
|  | Asian | 0.964 | 0.730 | 1.273 |  |
|  | Hispanic | **1.258** | **1.133** | **1.398** |  |
|  | Non-Hispanic White | 1.015 | 0.855 | 1.204 |  |
| HWD8 | African American | 1.065 | 0.637 | 1.780 | 0.519 |
|  | Asian | 0.949 | 0.615 | 1.463 |  |
|  | Hispanic | 1.083 | 0.924 | 1.269 |  |
|  | Non-Hispanic White | 0.868 | 0.670 | 1.125 |  |
| HWD9 | African American | 1.307 | 0.696 | 2.453 | 0.536 |
|  | Asian | 0.614 | 0.274 | 1.374 |  |
|  | Hispanic | 1.028 | 0.815 | 1.297 |  |
|  | Non-Hispanic White | 0.983 | 0.704 | 1.371 |  |
| HWD10 | African American | 1.480 | 0.733 | 2.988 | 0.735 |
|  | Asian | 0.917 | 0.475 | 1.772 |  |
|  | Hispanic | 0.988 | 0.787 | 1.240 |  |
|  | Non-Hispanic White | 1.002 | 0.711 | 1.414 |  |
| HWD11 | African American | 1.741 | 0.647 | 4.682 | 0.864 |
|  | Asian | 1.002 | 0.373 | 2.688 |  |
|  | Hispanic | 1.209 | 0.884 | 1.653 |  |
|  | Non-Hispanic White | 1.323 | 0.870 | 2.011 |  |
| HWD12 | African American | 0.675 | 0.094 | 4.830 | 0.950 |
|  | Asian | 1.093 | 0.273 | 4.385 |  |
|  | Hispanic | 1.167 | 0.748 | 1.821 |  |
|  | Non-Hispanic White | 1.037 | 0.552 | 1.947 |  |
| Education level | | | | | |
| HWD1 | < College | **1.182** | **1.107** | **1.261** | **0.034** |
|  | College | **1.077** | **1.026** | **1.129** |  |
|  | > College | 1.064 | 0.969 | 1.169 |  |
| HWD2 | < College | **1.205** | **1.122** | **1.295** | **0.040** |
|  | College | **1.078** | **1.022** | **1.138** |  |
|  | > College | **1.130** | **1.015** | **1.257** |  |
| HWD3 | < College | **1.246** | **1.149** | **1.350** | **0.029** |
|  | College | **1.096** | **1.029** | **1.166** |  |
|  | > College | 1.103 | 0.969 | 1.256 |  |
| HWD4 | < College | **1.248** | **1.143** | **1.362** | **0.035** |
|  | College | **1.100** | **1.026** | **1.178** |  |
|  | > College | 1.054 | 0.909 | 1.222 |  |

Note: Bold font face indicates a statistically significant result (*P* < 0.05). The P value refers to the comparison among subgroups and is obtained from Cochran’s Q test.

^a^ No PROM case more than 35 years old experienced HWD12 in this study.

**Table S7.** Adjusted hazard ratios (HRs) and 95% confidence intervals (CIs) of PROM associated with heatwaves among population subgroups stratified by maternal characteristics (continued).

| Description | | HRs | 95% CI | | *P* value for Cochran's Q test |
| --- | --- | --- | --- | --- | --- |
| Education level | | | | | |
| HWD5 | < College | **1.249** | **1.119** | **1.395** | 0.157 |
|  | College | **1.099** | **1.006** | **1.201** |  |
|  | > College | 1.126 | 0.932 | 1.361 |  |
| HWD6 | < College | **1.233** | **1.071** | **1.419** | 0.559 |
|  | College | **1.135** | **1.013** | **1.273** |  |
|  | > College | 1.154 | 0.901 | 1.477 |  |
| HWD7 | < College | **1.226** | **1.063** | **1.415** | 0.518 |
|  | College | 1.117 | 0.999 | 1.250 |  |
|  | > College | 1.194 | 0.940 | 1.518 |  |
| HWD8 | < College | 1.166 | 0.953 | 1.427 | 0.241 |
|  | College | 0.936 | 0.787 | 1.114 |  |
|  | > College | 0.967 | 0.650 | 1.439 |  |
| HWD9 | < College | **1.337** | **1.030** | **1.735** | **0.028** |
|  | College | 0.843 | 0.649 | 1.096 |  |
|  | > College | 0.765 | 0.409 | 1.430 |  |
| HWD10 | < College | 1.123 | 0.839 | 1.503 | 0.709 |
|  | College | 1.003 | 0.789 | 1.275 |  |
|  | > College | 0.933 | 0.560 | 1.556 |  |
| HWD11 | < College | **1.742** | **1.209** | **2.510** | 0.127 |
|  | College | 1.059 | 0.755 | 1.486 |  |
|  | > College | 1.182 | 0.611 | 2.287 |  |
| HWD12 | < College | **1.883** | **1.178** | **3.012** | **0.037** |
|  | College | 0.759 | 0.439 | 1.315 |  |
|  | > College | 1.242 | 0.463 | 3.332 |  |
| Income level | | | | | |
| HWD1 | < 50^th^ | **1.136** | **1.079** | **1.195** | **0.033** |
|  | ≥ 50^th^ | **1.052** | **1.001** | **1.106** |  |
| HWD2 | < 50^th^ | **1.131** | **1.068** | **1.197** | 0.373 |
|  | ≥ 50^th^ | **1.092** | **1.033** | **1.155** |  |
| HWD3 | < 50^th^ | **1.127** | **1.056** | **1.203** | 0.858 |
|  | ≥ 50^th^ | **1.130** | **1.059** | **1.206** |  |
| HWD4 | < 50^th^ | **1.149** | **1.070** | **1.233** | 0.452 |
|  | ≥ 50^th^ | **1.107** | **1.030** | **1.191** |  |
| HWD5 | < 50^th^ | **1.140** | **1.042** | **1.247** | 0.745 |
|  | ≥ 50^th^ | **1.133** | **1.032** | **1.243** |  |
| HWD6 | < 50^th^ | 1.120 | 0.997 | 1.257 | 0.424 |
|  | ≥ 50^th^ | **1.194** | **1.060** | **1.346** |  |
| HWD7 | < 50^th^ | **1.161** | **1.036** | **1.301** | 0.711 |
|  | ≥ 50^th^ | **1.131** | **1.003** | **1.275** |  |
| HWD8 | < 50^th^ | 1.046 | 0.888 | 1.234 | 0.491 |
|  | ≥ 50^th^ | 0.961 | 0.795 | 1.161 |  |
| HWD9 | < 50^th^ | 1.025 | 0.816 | 1.288 | 0.748 |
|  | ≥ 50^th^ | 0.968 | 0.734 | 1.276 |  |
| HWD10 | < 50^th^ | 0.951 | 0.752 | 1.203 | 0.381 |
|  | ≥ 50^th^ | 1.106 | 0.854 | 1.431 |  |
| HWD11 | < 50^th^ | 1.113 | 0.821 | 1.507 | 0.174 |
|  | ≥ 50^th^ | **1.539** | **1.074** | **2.205** |  |
| HWD12 | < 50^th^ | 0.933 | 0.604 | 1.441 | 0.121 |
|  | ≥ 50^th^ | 1.583 | 0.947 | 2.646 |  |

Note: Bold font face indicates a statistically significant result (*P* < 0.05). The P value refers to the comparison among subgroups and is obtained from Cochran’s Q test.

^a^ No PROM case more than 35 years old experienced HWD12 in this study.

**Table S7.** Adjusted hazard ratios (HRs) and 95% confidence intervals (CIs) of PROM associated with heatwaves among population subgroups stratified by maternal characteristics (continued).

| Description | | HRs | 95% CI | | *P* value for Cochran's Q test |
| --- | --- | --- | --- | --- | --- |
| Smoking | | | | | |
| HWD1 | Smoker | **1.162** | **1.068** | **1.265** | 0.118 |
|  | Non-smoker | **1.081** | **1.039** | **1.124** |  |
| HWD2 | Smoker | **1.207** | **1.099** | **1.326** | 0.062 |
|  | Non-smoker | **1.096** | **1.048** | **1.145** |  |
| HWD3 | Smoker | **1.202** | **1.078** | **1.342** | 0.230 |
|  | Non-smoker | **1.120** | **1.065** | **1.178** |  |
| HWD4 | Smoker | **1.275** | **1.132** | **1.436** | **0.029** |
|  | Non-smoker | **1.104** | **1.044** | **1.168** |  |
| HWD5 | Smoker | **1.341** | **1.152** | **1.561** | **0.018** |
|  | Non-smoker | **1.098** | **1.023** | **1.179** |  |
| HWD6 | Smoker | **1.464** | **1.208** | **1.774** | **0.007** |
|  | Non-smoker | 1.091 | 0.995 | 1.196 |  |
| HWD7 | Smoker | 1.216 | 0.991 | 1.493 | 0.517 |
|  | Non-smoker | **1.132** | **1.035** | **1.239** |  |
| HWD8 | Smoker | 1.179 | 0.864 | 1.610 | 0.261 |
|  | Non-smoker | 0.971 | 0.849 | 1.112 |  |
| HWD9 | Smoker | 1.164 | 0.739 | 1.834 | 0.468 |
|  | Non-smoker | 0.970 | 0.802 | 1.174 |  |
| HWD10 | Smoker | 1.202 | 0.753 | 1.918 | 0.420 |
|  | Non-smoker | 0.978 | 0.811 | 1.179 |  |
| HWD11 | Smoker | 0.961 | 0.430 | 2.149 | 0.508 |
|  | Non-smoker | **1.276** | **1.001** | **1.626** |  |
| HWD12 | Smoker | 1.000 | 0.322 | 3.108 | 0.835 |
|  | Non-smoker | 1.132 | 0.800 | 1.602 |  |

Note: Bold font face indicates a statistically significant result (*P* < 0.05). The P value refers to the comparison among subgroups and is obtained from Cochran’s Q test.

^a^ No PROM case more than 35 years old experienced HWD12 in this study.

**Table S8.** Adjusted hazard ratios (HRs) and 95% confidence intervals (CIs) of PROM associated with heatwaves of sensitivity analyses.

| Heatwave definitions | Models | | | | | |  |
| --- | --- | --- | --- | --- | --- | --- | --- |
|  | 4 days exposure | 2 weeks exposure | Max temp model ^a^ | Medical center ^b^ | County ^c^ | Discrete time model ^d^ | |
| HWD1 | **1.071 (1.030, 1.115)** | **1.205 (1.165, 1.245)** | **1.060 (1.020, 1.101)** | **1.051 (1.012, 1.092)** | **1.057 (1.020,1.096)** | **1.044 (1.006, 1.083)** | |
| HWD2 | **1.104 (1.054, 1.157)** | **1.174 (1.134, 1.216)** | **1.080 (1.035, 1.127)** | **1.079 (1.034, 1.127)** | **1.072 (1.029,1.116)** | **1.049 (1.006, 1.093)** | |
| HWD3 | **1.116 (1.055, 1.180)** | **1.194 (1.150, 1.241)** | **1.079 (1.026, 1.136)** | **1.096 (1.043, 1.152)** | **1.085 (1.035,1.136)** | **1.053 (1.004, 1.104)** | |
| HWD4 | **1.145 (1.078, 1.216)** | **1.174 (1.126, 1.224)** | **1.068 (1.008, 1.130)** | **1.090 (1.032, 1.151)** | **1.086 (1.032,1.142)** | **1.060 (1.006, 1.117)** | |
| HWD5 | **1.133 (1.044, 1.231)** | **1.185 (1.127, 1.246)** | **1.086 (1.007, 1.172)** | **1.093 (1.018, 1.173)** | **1.082 (1.014,1.155)** | **1.070 (1.001, 1.143)** | |
| HWD6 | **1.130 (1.008, 1.266)** | **1.202 (1.130, 1.278)** | 1.091 (0.986, 1.207) | **1.136 (1.035, 1.246)** | **1.087 (1.000,1.182)** | 1.075 (0.987, 1.171) | |
| HWD7 | 1.100 (0.990, 1.222) | **1.192 (1.120, 1.269)** | **1.111 (1.027, 1.201)** | **1.095 (1.000, 1.200)** | **1.093 (1.007,1.187)** | 1.064 (0.977, 1.157) | |
| HWD8 | 1.049 (0.894, 1.232) | **1.128 (1.033, 1.231)** | 1.114 (0.988, 1.254) | 0.964 (0.834, 1.116) | 0.950 (0.839,1.075) | 0.920 (0.810, 1.044) | |
| HWD9 | 1.056 (0.841, 1.326) | 1.122 (0.993, 1.269) | 1.068 (0.904, 1.262) | 0.940 (0.751, 1.177) | 0.935 (0.785,1.114) | 0.917 (0.766, 1.098) | |
| HWD10 | 1.028 (0.828, 1.277) | 1.094 (0.964, 1.241) | 1.119 (0.977, 1.281) | 0.998 (0.807, 1.235) | 0.944 (0.795,1.122) | 0.907 (0.760, 1.083) | |
| HWD11 | 1.134 (0.830, 1.548) | **1.244 (1.041, 1.486)** | 1.248 (0.992, 1.569) | 1.289 (0.939, 1.769) | 1.141 (0.908,1.435) | 1.133 (0.895, 1.435) | |
| HWD12 | 1.102 (0.681, 1.782) | 1.114 (0.874, 1.420) | 1.321 (0.982, 1.776) | 1.322 (0.819, 2.133) | 1.015 (0.731,1.411) | 1.034 (0.737, 1.451) | |

Note: Models are adjusted for maternal age, race/ethnicity, education level, median family household income, pre-pregnancy BMI, smoking status, year of infant birth, and parity. Bold font face indicates a statistically significant result (*P* < 0.05).

^a^ Model using heatwave definitions based on daily maximum temperature.

^b^ Medical center is fitted as a random effect.

^c^ County of residence is fitted as a random effect.

^d^ Model using the discrete time approach with the random effect of county.

**Table S9.** The effect modification in associations between heatwaves and PROM by exposure to air pollution during the entire pregnancy estimated in models with the interaction term.

| Description | | HRs | 95% CI | | *P* value for the interaction |
| --- | --- | --- | --- | --- | --- |
| PM_2.5_ | | | | | |
| HWD1 | < 50^th^ | **1.070** | **1.017** | **1.125** | 0.184 |
|  | ≥ 50^th^ | **1.124** | **1.064** | **1.188** |  |
| HWD2 | < 50^th^ | **1.095** | **1.035** | **1.158** | 0.387 |
|  | ≥ 50^th^ | **1.136** | **1.068** | **1.208** |  |
| HWD3 | < 50^th^ | **1.107** | **1.037** | **1.181** | 0.463 |
|  | ≥ 50^th^ | **1.147** | **1.068** | **1.232** |  |
| HWD4 | < 50^th^ | **1.115** | **1.040** | **1.195** | 0.367 |
|  | ≥ 50^th^ | **1.171** | **1.079** | **1.270** |  |
| HWD5 | < 50^th^ | **1.132** | **1.036** | **1.237** | 0.718 |
|  | ≥ 50^th^ | **1.160** | **1.046** | **1.288** |  |
| HWD6 | < 50^th^ | 1.114 | 0.996 | 1.246 | 0.191 |
|  | ≥ 50^th^ | **1.253** | **1.093** | **1.436** |  |
| HWD7 | < 50^th^ | 1.103 | 0.984 | 1.238 | 0.262 |
|  | ≥ 50^th^ | **1.219** | **1.069** | **1.390** |  |
| HWD8 | < 50^th^ | 0.957 | 0.807 | 1.134 | 0.530 |
|  | ≥ 50^th^ | 1.042 | 0.847 | 1.282 |  |
| HWD9 | < 50^th^ | 0.902 | 0.715 | 1.139 | 0.163 |
|  | ≥ 50^th^ | 1.185 | 0.873 | 1.609 |  |
| HWD10 | < 50^th^ | 0.970 | 0.774 | 1.215 | 0.672 |
|  | ≥ 50^th^ | 1.056 | 0.766 | 1.455 |  |
| HWD11 | < 50^th^ | 1.170 | 0.872 | 1.569 | 0.461 |
|  | ≥ 50^th^ | 1.438 | 0.903 | 2.290 |  |
| HWD12 | < 50^th^ | 1.132 | 0.759 | 1.689 | 0.987 |
|  | ≥ 50^th^ | 1.140 | 0.543 | 2.393 |  |
| NO_2_ | | | | | |
| HWD1 | < 50^th^ | **1.130** | **1.075** | **1.189** | 0.075 |
|  | ≥ 50^th^ | 1.057 | 1.000 | 1.117 |  |
| HWD2 | < 50^th^ | **1.145** | **1.083** | **1.210** | 0.161 |
|  | ≥ 50^th^ | **1.079** | **1.012** | **1.149** |  |
| HWD3 | < 50^th^ | **1.144** | **1.074** | **1.218** | 0.491 |
|  | ≥ 50^th^ | **1.105** | **1.026** | **1.191** |  |
| HWD4 | < 50^th^ | **1.142** | **1.067** | **1.222** | 0.962 |
|  | ≥ 50^th^ | **1.139** | **1.047** | **1.238** |  |
| HWD5 | < 50^th^ | **1.168** | **1.074** | **1.272** | 0.477 |
|  | ≥ 50^th^ | 1.111 | 0.993 | 1.243 |  |
| HWD6 | < 50^th^ | **1.137** | **1.023** | **1.265** | 0.354 |
|  | ≥ 50^th^ | **1.241** | **1.067** | **1.443** |  |
| HWD7 | < 50^th^ | **1.141** | **1.023** | **1.272** | 0.737 |
|  | ≥ 50^th^ | **1.177** | **1.020** | **1.357** |  |
| HWD8 | < 50^th^ | 0.979 | 0.837 | 1.144 | 0.744 |
|  | ≥ 50^th^ | 1.027 | 0.805 | 1.311 |  |
| HWD9 | < 50^th^ | 0.966 | 0.785 | 1.188 | 0.547 |
|  | ≥ 50^th^ | 1.112 | 0.737 | 1.678 |  |
| HWD10 | < 50^th^ | 0.971 | 0.785 | 1.202 | 0.545 |
|  | ≥ 50^th^ | 1.107 | 0.767 | 1.598 |  |
| HWD11 | < 50^th^ | 1.213 | 0.930 | 1.583 | 0.589 |
|  | ≥ 50^th^ | 1.489 | 0.743 | 2.983 |  |
| HWD12 | < 50^th^ | 1.147 | 0.797 | 1.650 | 0.915 |
|  | ≥ 50^th^ | 1.061 | 0.268 | 4.195 |  |

Note: Models are adjusted for maternal age, race/ethnicity, education level, median family household income, pre-pregnancy BMI, smoking status, year of infant birth, and parity. The cutoff concentrations of PM_2.5_ and NO_2_ are 11.26 µg/m^3^ and 16.45 ppb, respectively. Bold font face indicates a statistically significant result (*P* < 0.05). The *P* value is for the interaction term between the heatwave exposure variable and the effect modifier.

**Table S10.** The effect modification in associations between heatwaves and PROM by exposure to air pollution during the last three months of pregnancy estimated in models with the interaction term.

| Description | | HRs | 95% CI | | *P* value for the interaction |
| --- | --- | --- | --- | --- | --- |
| PM_2.5_ | | | | | |
| HWD1 | < 50^th^ | 1.052 | 0.998 | 1.108 | **0.041** |
|  | ≥ 50^th^ | **1.133** | **1.078** | **1.190** |  |
| HWD2 | < 50^th^ | 1.057 | 0.996 | 1.121 | **0.017** |
|  | ≥ 50^th^ | **1.164** | **1.102** | **1.230** |  |
| HWD3 | < 50^th^ | **1.103** | **1.032** | **1.180** | 0.370 |
|  | ≥ 50^th^ | **1.151** | **1.079** | **1.227** |  |
| HWD4 | < 50^th^ | **1.099** | **1.022** | **1.182** | 0.331 |
|  | ≥ 50^th^ | **1.156** | **1.076** | **1.242** |  |
| HWD5 | < 50^th^ | **1.148** | **1.046** | **1.259** | 0.874 |
|  | ≥ 50^th^ | **1.136** | **1.037** | **1.245** |  |
| HWD6 | < 50^th^ | **1.159** | **1.030** | **1.303** | 0.929 |
|  | ≥ 50^th^ | **1.168** | **1.036** | **1.316** |  |
| HWD7 | < 50^th^ | **1.174** | **1.047** | **1.317** | 0.677 |
|  | ≥ 50^th^ | **1.134** | **1.005** | **1.279** |  |
| HWD8 | < 50^th^ | 1.051 | 0.882 | 1.253 | 0.458 |
|  | ≥ 50^th^ | 0.956 | 0.798 | 1.145 |  |
| HWD9 | < 50^th^ | 1.046 | 0.815 | 1.343 | 0.631 |
|  | ≥ 50^th^ | 0.959 | 0.744 | 1.236 |  |
| HWD10 | < 50^th^ | 1.010 | 0.807 | 1.266 | 0.964 |
|  | ≥ 50^th^ | 1.019 | 0.770 | 1.348 |  |
| HWD11 | < 50^th^ | 1.261 | 0.926 | 1.717 | 0.942 |
|  | ≥ 50^th^ | 1.239 | 0.859 | 1.788 |  |
| HWD12 | < 50^th^ | 1.229 | 0.823 | 1.834 | 0.619 |
|  | ≥ 50^th^ | 1.019 | 0.547 | 1.898 |  |
| NO_2_ | | | | | |
| HWD1 | < 50^th^ | **1.099** | **1.045** | **1.156** | 0.899 |
|  | ≥ 50^th^ | **1.104** | **1.049** | **1.161** |  |
| HWD2 | < 50^th^ | **1.114** | **1.053** | **1.178** | 0.807 |
|  | ≥ 50^th^ | **1.125** | **1.061** | **1.192** |  |
| HWD3 | < 50^th^ | **1.121** | **1.052** | **1.195** | 0.590 |
|  | ≥ 50^th^ | **1.150** | **1.074** | **1.231** |  |
| HWD4 | < 50^th^ | **1.126** | **1.050** | **1.208** | 0.810 |
|  | ≥ 50^th^ | **1.140** | **1.058** | **1.230** |  |
| HWD5 | < 50^th^ | **1.173** | **1.077** | **1.278** | 0.498 |
|  | ≥ 50^th^ | **1.121** | **1.013** | **1.239** |  |
| HWD6 | < 50^th^ | **1.171** | **1.052** | **1.303** | 0.968 |
|  | ≥ 50^th^ | **1.175** | **1.027** | **1.344** |  |
| HWD7 | < 50^th^ | **1.197** | **1.074** | **1.334** | 0.379 |
|  | ≥ 50^th^ | 1.110 | 0.975 | 1.264 |  |
| HWD8 | < 50^th^ | 1.008 | 0.859 | 1.183 | 0.948 |
|  | ≥ 50^th^ | 1.017 | 0.829 | 1.247 |  |
| HWD9 | < 50^th^ | 0.973 | 0.781 | 1.213 | 0.489 |
|  | ≥ 50^th^ | 1.110 | 0.821 | 1.501 |  |
| HWD10 | < 50^th^ | 0.989 | 0.789 | 1.241 | 0.713 |
|  | ≥ 50^th^ | 1.058 | 0.802 | 1.397 |  |
| HWD11 | < 50^th^ | 1.274 | 0.958 | 1.694 | 0.937 |
|  | ≥ 50^th^ | 1.248 | 0.819 | 1.902 |  |
| HWD12 | < 50^th^ | 1.215 | 0.832 | 1.772 | 0.702 |
|  | ≥ 50^th^ | 1.033 | 0.492 | 2.168 |  |

Note: Models are adjusted for maternal age, race/ethnicity, education level, median family household income, pre-pregnancy BMI, smoking status, year of infant birth, and parity. The cutoff concentrations of PM_2.5_ and NO_2_ are 10.98 µg/m^3^ and 11.76 ppb, respectively. Bold font face indicates a statistically significant result (*P* < 0.05). The *P* value is for the interaction term between the heatwave exposure variable and the effect modifier.

**Table S11.** The effect modification in associations between heatwaves and PROM by exposure to air pollution during the last month of pregnancy estimated in models with the interaction term.

| Description | | HRs | 95% CI | | *P* value for the interaction |
| --- | --- | --- | --- | --- | --- |
| PM_2.5_ | | | | | |
| HWD1 | < 50^th^ | 1.045 | 0.989 | 1.104 | 0.081 |
|  | ≥ 50^th^ | **1.114** | **1.063** | **1.168** |  |
| HWD2 | < 50^th^ | **1.067** | **1.002** | **1.136** | 0.184 |
|  | ≥ 50^th^ | **1.127** | **1.070** | **1.187** |  |
| HWD3 | < 50^th^ | **1.124** | **1.044** | **1.209** | 0.859 |
|  | ≥ 50^th^ | **1.114** | **1.050** | **1.183** |  |
| HWD4 | < 50^th^ | **1.095** | **1.012** | **1.185** | 0.415 |
|  | ≥ 50^th^ | **1.143** | **1.069** | **1.222** |  |
| HWD5 | < 50^th^ | **1.157** | **1.045** | **1.280** | 0.660 |
|  | ≥ 50^th^ | **1.123** | **1.032** | **1.222** |  |
| HWD6 | < 50^th^ | **1.169** | **1.027** | **1.330** | 0.852 |
|  | ≥ 50^th^ | **1.150** | **1.031** | **1.283** |  |
| HWD7 | < 50^th^ | **1.202** | **1.062** | **1.362** | 0.371 |
|  | ≥ 50^th^ | 1.115 | 0.998 | 1.245 |  |
| HWD8 | < 50^th^ | 1.010 | 0.828 | 1.232 | 0.935 |
|  | ≥ 50^th^ | 0.999 | 0.850 | 1.173 |  |
| HWD9 | < 50^th^ | 1.101 | 0.845 | 1.434 | 0.317 |
|  | ≥ 50^th^ | 0.917 | 0.721 | 1.167 |  |
| HWD10 | < 50^th^ | 1.097 | 0.860 | 1.398 | 0.388 |
|  | ≥ 50^th^ | 0.940 | 0.731 | 1.210 |  |
| HWD11 | < 50^th^ | 1.329 | 0.961 | 1.839 | 0.681 |
|  | ≥ 50^th^ | 1.205 | 0.859 | 1.691 |  |
| HWD12 | < 50^th^ | 1.260 | 0.838 | 1.895 | 0.552 |
|  | ≥ 50^th^ | 1.013 | 0.559 | 1.833 |  |
| NO_2_ | | | | | |
| HWD1 | < 50^th^ | **1.086** | **1.032** | **1.142** | 0.515 |
|  | ≥ 50^th^ | **1.111** | **1.057** | **1.169** |  |
| HWD2 | < 50^th^ | **1.088** | **1.028** | **1.151** | 0.200 |
|  | ≥ 50^th^ | **1.145** | **1.083** | **1.212** |  |
| HWD3 | < 50^th^ | **1.108** | **1.039** | **1.182** | 0.321 |
|  | ≥ 50^th^ | **1.161** | **1.086** | **1.240** |  |
| HWD4 | < 50^th^ | **1.123** | **1.047** | **1.205** | 0.683 |
|  | ≥ 50^th^ | **1.147** | **1.065** | **1.235** |  |
| HWD5 | < 50^th^ | **1.148** | **1.051** | **1.254** | 0.952 |
|  | ≥ 50^th^ | **1.152** | **1.047** | **1.268** |  |
| HWD6 | < 50^th^ | **1.157** | **1.036** | **1.293** | 0.772 |
|  | ≥ 50^th^ | **1.186** | **1.045** | **1.347** |  |
| HWD7 | < 50^th^ | **1.174** | **1.052** | **1.311** | 0.714 |
|  | ≥ 50^th^ | **1.138** | **1.003** | **1.291** |  |
| HWD8 | < 50^th^ | 0.989 | 0.838 | 1.167 | 0.664 |
|  | ≥ 50^th^ | 1.046 | 0.864 | 1.267 |  |
| HWD9 | < 50^th^ | 0.962 | 0.770 | 1.202 | 0.452 |
|  | ≥ 50^th^ | 1.106 | 0.824 | 1.486 |  |
| HWD10 | < 50^th^ | 1.005 | 0.804 | 1.256 | 0.856 |
|  | ≥ 50^th^ | 1.039 | 0.783 | 1.379 |  |
| HWD11 | < 50^th^ | 1.320 | 0.994 | 1.751 | 0.693 |
|  | ≥ 50^th^ | 1.191 | 0.782 | 1.816 |  |
| HWD12 | < 50^th^ | 1.222 | 0.837 | 1.784 | 0.627 |
|  | ≥ 50^th^ | 0.994 | 0.474 | 2.087 |  |

Note: Models are adjusted for maternal age, race/ethnicity, education level, median family household income, pre-pregnancy BMI, smoking status, year of infant birth, and parity. The cutoff concentrations of PM_2.5_ and NO_2_ are 11.39 µg/m^3^ and 11.37 ppb, respectively. Bold font face indicates a statistically significant result (*P* < 0.05). The *P* value is for the interaction term between the heatwave exposure variable and the effect modifier.

**Table S12.** The effect modification in associations between heatwaves and PROM by climate adaptation measures estimated in models with the interaction term.

| Description | | HRs | 95% CI | | *P* value of the interaction |
| --- | --- | --- | --- | --- | --- |
| Green space | | | | | |
| HWD1 | < 50^th^ | **1.097** | **1.043** | **1.154** | 0.658 |
|  | ≥ 50^th^ | **1.080** | **1.028** | **1.135** |  |
| HWD2 | < 50^th^ | **1.129** | **1.068** | **1.194** | 0.310 |
|  | ≥ 50^th^ | **1.084** | **1.025** | **1.147** |  |
| HWD3 | < 50^th^ | **1.164** | **1.093** | **1.241** | 0.115 |
|  | ≥ 50^th^ | **1.082** | **1.013** | **1.157** |  |
| HWD4 | < 50^th^ | **1.165** | **1.086** | **1.249** | 0.119 |
|  | ≥ 50^th^ | 1.076 | 1.000 | 1.158 |  |
| HWD5 | < 50^th^ | **1.188** | **1.088** | **1.296** | 0.088 |
|  | ≥ 50^th^ | 1.062 | 0.965 | 1.169 |  |
| HWD6 | < 50^th^ | **1.220** | **1.094** | **1.361** | 0.112 |
|  | ≥ 50^th^ | 1.066 | 0.937 | 1.212 |  |
| HWD7 | < 50^th^ | **1.230** | **1.103** | **1.371** | 0.065 |
|  | ≥ 50^th^ | 1.053 | 0.929 | 1.194 |  |
| HWD8 | < 50^th^ | 1.090 | 0.932 | 1.274 | 0.102 |
|  | ≥ 50^th^ | 0.881 | 0.718 | 1.080 |  |
| HWD9 | < 50^th^ | 1.101 | 0.892 | 1.360 | 0.164 |
|  | ≥ 50^th^ | 0.842 | 0.613 | 1.155 |  |
| HWD10 | < 50^th^ | 1.103 | 0.894 | 1.362 | 0.173 |
|  | ≥ 50^th^ | 0.854 | 0.629 | 1.159 |  |
| HWD11 | < 50^th^ | **1.376** | **1.045** | **1.811** | 0.299 |
|  | ≥ 50^th^ | 1.051 | 0.683 | 1.617 |  |
| HWD12 | < 50^th^ | 1.199 | 0.821 | 1.750 | 0.613 |
|  | ≥ 50^th^ | 0.978 | 0.488 | 1.962 |  |
| AC penetration rate | | | | | |
| HWD1 | < 50^th^ | 1.076 | 0.995 | 1.163 | 0.514 |
|  | ≥ 50^th^ | 1.038 | 0.963 | 1.119 |  |
| HWD2 | < 50^th^ | **1.123** | **1.033** | **1.221** | 0.408 |
|  | ≥ 50^th^ | 1.070 | 0.988 | 1.159 |  |
| HWD3 | < 50^th^ | **1.148** | **1.045** | **1.261** | 0.373 |
|  | ≥ 50^th^ | 1.083 | 0.991 | 1.182 |  |
| HWD4 | < 50^th^ | **1.166** | **1.051** | **1.294** | 0.239 |
|  | ≥ 50^th^ | 1.071 | 0.971 | 1.181 |  |
| HWD5 | < 50^th^ | **1.152** | **1.010** | **1.313** | 0.532 |
|  | ≥ 50^th^ | 1.088 | 0.964 | 1.229 |  |
| HWD6 | < 50^th^ | 1.095 | 0.920 | 1.304 | 0.521 |
|  | ≥ 50^th^ | **1.181** | **1.016** | **1.372** |  |
| HWD7 | < 50^th^ | 1.149 | 0.971 | 1.359 | 0.639 |
|  | ≥ 50^th^ | 1.088 | 0.934 | 1.268 |  |
| HWD8 | < 50^th^ | 1.095 | 0.851 | 1.409 | 0.550 |
|  | ≥ 50^th^ | 0.989 | 0.795 | 1.232 |  |
| HWD9 | < 50^th^ | 1.205 | 0.852 | 1.705 | 0.366 |
|  | ≥ 50^th^ | 0.974 | 0.714 | 1.328 |  |
| HWD10 | < 50^th^ | 1.297 | 0.902 | 1.865 | 0.164 |
|  | ≥ 50^th^ | 0.922 | 0.671 | 1.267 |  |
| HWD11 | < 50^th^ | 1.562 | 0.950 | 2.568 | 0.368 |
|  | ≥ 50^th^ | 1.145 | 0.721 | 1.819 |  |
| HWD12 | < 50^th^ | 1.473 | 0.696 | 3.117 | 0.387 |
|  | ≥ 50^th^ | 0.949 | 0.488 | 1.844 |  |

Note: Models are adjusted for maternal age, race/ethnicity, education level, median family household income, pre-pregnancy BMI, smoking status, year of infant birth, and parity. The cutoff points of subgroups are 24.24% and 0.82 for green space and AC penetration rates, respectively. Bold font face indicates a statistically significant result (*P* < 0.05). The *P* value is for the interaction term between the heatwave exposure variable and the effect modifier.

**Table S13.** The effect modification in associations between heatwaves and PROM by maternal characteristics estimated in models with the interaction term.

| Description | | HRs | 95% CI | | *P* value for the interaction |
| --- | --- | --- | --- | --- | --- |
| Maternal Age | | | | | |
| HWD1 | < 25 | **1.159** | **1.075** | **1.250** | 0.148 |
|  | 25-34 | **1.073** | **1.026** | **1.123** |  |
|  | ≥ 35 | 1.053 | 0.973 | 1.139 |  |
| HWD2 | < 25 | **1.217** | **1.121** | **1.322** | **0.032** |
|  | 25-34 | **1.074** | **1.021** | **1.130** |  |
|  | ≥ 35 | 1.080 | 0.988 | 1.182 |  |
| HWD3 | < 25 | **1.230** | **1.121** | **1.350** | 0.089 |
|  | 25-34 | **1.096** | **1.033** | **1.163** |  |
|  | ≥ 35 | 1.084 | 0.975 | 1.206 |  |
| HWD4 | < 25 | **1.251** | **1.130** | **1.386** | 0.054 |
|  | 25-34 | **1.087** | **1.019** | **1.161** |  |
|  | ≥ 35 | 1.076 | 0.957 | 1.210 |  |
| HWD5 | < 25 | **1.278** | **1.125** | **1.451** | 0.064 |
|  | 25-34 | 1.065 | 0.979 | 1.160 |  |
|  | ≥ 35 | 1.133 | 0.977 | 1.315 |  |
| HWD6 | < 25 | **1.357** | **1.159** | **1.588** | **0.047** |
|  | 25-34 | 1.072 | 0.960 | 1.196 |  |
|  | ≥ 35 | 1.094 | 0.893 | 1.340 |  |
| HWD7 | < 25 | **1.299** | **1.105** | **1.526** | 0.169 |
|  | 25-34 | 1.096 | 0.984 | 1.221 |  |
|  | ≥ 35 | 1.063 | 0.874 | 1.293 |  |
| HWD8 | < 25 | 1.215 | 0.970 | 1.522 | 0.126 |
|  | 25-34 | 0.926 | 0.786 | 1.092 |  |
|  | ≥ 35 | 0.904 | 0.656 | 1.246 |  |
| HWD9 | < 25 | **1.383** | **1.024** | **1.867** | **0.031** |
|  | 25-34 | 0.904 | 0.714 | 1.145 |  |
|  | ≥ 35 | 0.713 | 0.429 | 1.186 |  |
| HWD10 | < 25 | 1.256 | 0.923 | 1.708 | 0.082 |
|  | 25-34 | 0.980 | 0.783 | 1.226 |  |
|  | ≥ 35 | 0.624 | 0.362 | 1.078 |  |
| HWD11 | < 25 | **1.923** | **1.310** | **2.822** | **0.026** |
|  | 25-34 | 1.114 | 0.816 | 1.521 |  |
|  | ≥ 35 | 0.733 | 0.349 | 1.540 |  |
| HWD12 | < 25 | **2.010** | **1.241** | **3.256** | **0.031** |
|  | 25-34 | 0.976 | 0.620 | 1.537 |  |
|  | ≥ 35^a^ | - | - | - |  |
| Maternal race/ethnicity | | | | | |
| HWD1 | African American | 1.049 | 0.915 | 1.203 | 0.900 |
|  | Asian | 1.068 | 0.968 | 1.179 |  |
|  | Hispanic | **1.091** | **1.039** | **1.144** |  |
|  | Non-Hispanic White | **1.103** | **1.030** | **1.182** |  |
| HWD2 | African American | 1.016 | 0.870 | 1.187 | 0.670 |
|  | Asian | 1.105 | 0.987 | 1.238 |  |
|  | Hispanic | **1.110** | **1.052** | **1.171** |  |
|  | Non-Hispanic White | **1.134** | **1.051** | **1.224** |  |
| HWD3 | African American | 1.007 | 0.839 | 1.208 | 0.674 |
|  | Asian | 1.131 | 0.987 | 1.296 |  |
|  | Hispanic | **1.130** | **1.063** | **1.202** |  |
|  | Non-Hispanic White | **1.139** | **1.043** | **1.244** |  |

Note: Bold font face indicates a statistically significant result (*P* < 0.05). The *P* value is for the interaction term between the heatwave exposure variable and the effect modifier.

^a^ No PROM case more than 35 years old experienced HWD12 in this study.

**Table S13.** The effect modification in associations between heatwaves and PROM by maternal characteristics estimated in models with the interaction term (continued).

| Description | | HRs | 95% CI | | *P* value for the interaction |
| --- | --- | --- | --- | --- | --- |
| Maternal race/ethnicity | | | | | |
| HWD4 | African American | 0.884 | 0.712 | 1.098 | 0.147 |
|  | Asian | 1.110 | 0.956 | 1.290 |  |
|  | Hispanic | **1.153** | **1.078** | **1.234** |  |
|  | Non-Hispanic White | **1.135** | **1.030** | **1.251** |  |
| HWD5 | African American | 1.009 | 0.765 | 1.331 | 0.809 |
|  | Asian | 1.098 | 0.901 | 1.337 |  |
|  | Hispanic | **1.154** | **1.059** | **1.256** |  |
|  | Non-Hispanic White | 1.124 | 0.991 | 1.274 |  |
| HWD6 | African American | 1.167 | 0.837 | 1.629 | 0.988 |
|  | Asian | 1.095 | 0.837 | 1.433 |  |
|  | Hispanic | **1.152** | **1.032** | **1.286** |  |
|  | Non-Hispanic White | 1.142 | 0.974 | 1.340 |  |
| HWD7 | African American | 1.154 | 0.820 | 1.626 | 0.119 |
|  | Asian | 0.947 | 0.718 | 1.248 |  |
|  | Hispanic | **1.236** | **1.113** | **1.372** |  |
|  | Non-Hispanic White | 1.015 | 0.856 | 1.203 |  |
| HWD8 | African American | 1.039 | 0.624 | 1.730 | 0.583 |
|  | Asian | 0.911 | 0.592 | 1.402 |  |
|  | Hispanic | 1.061 | 0.906 | 1.242 |  |
|  | Non-Hispanic White | 0.867 | 0.671 | 1.120 |  |
| HWD9 | African American | 1.303 | 0.699 | 2.430 | 0.500 |
|  | Asian | 0.591 | 0.266 | 1.316 |  |
|  | Hispanic | 1.002 | 0.795 | 1.264 |  |
|  | Non-Hispanic White | 0.972 | 0.698 | 1.353 |  |
| HWD10 | African American | 1.338 | 0.668 | 2.683 | 0.819 |
|  | Asian | 0.868 | 0.451 | 1.671 |  |
|  | Hispanic | 0.963 | 0.768 | 1.208 |  |
|  | Non-Hispanic White | 0.985 | 0.701 | 1.385 |  |
| HWD11 | African American | 1.687 | 0.633 | 4.498 | 0.867 |
|  | Asian | 0.965 | 0.363 | 2.569 |  |
|  | Hispanic | 1.188 | 0.869 | 1.623 |  |
|  | Non-Hispanic White | 1.284 | 0.848 | 1.943 |  |
| HWD12 | African American | 0.664 | 0.097 | 4.557 | 0.951 |
|  | Asian | 1.051 | 0.267 | 4.141 |  |
|  | Hispanic | 1.137 | 0.729 | 1.773 |  |
|  | Non-Hispanic White | 1.005 | 0.535 | 1.885 |  |
| Education level | | | | | |
| HWD1 | < College | **1.160** | **1.088** | **1.237** | 0.064 |
|  | College | **1.069** | **1.020** | **1.121** |  |
|  | > College | 1.038 | 0.946 | 1.139 |  |
| HWD2 | < College | **1.184** | **1.103** | **1.270** | 0.076 |
|  | College | **1.071** | **1.016** | **1.130** |  |
|  | > College | 1.095 | 0.985 | 1.218 |  |
| HWD3 | < College | **1.226** | **1.132** | **1.327** | **0.035** |
|  | College | **1.086** | **1.021** | **1.155** |  |
|  | > College | 1.062 | 0.934 | 1.208 |  |
| HWD4 | < College | **1.224** | **1.122** | **1.334** | **0.036** |
|  | College | **1.091** | **1.019** | **1.168** |  |
|  | > College | 1.009 | 0.871 | 1.169 |  |

Note: Bold font face indicates a statistically significant result (*P* < 0.05). The *P* value is for the interaction term between the heatwave exposure variable and the effect modifier.

^a^ No PROM case more than 35 years old experienced HWD12 in this study.

**Table S13.** The effect modification in associations between heatwaves and PROM by maternal characteristics estimated in models with the interaction term (continued).

| Description | | HRs | 95% CI | | *P* value for the interaction |
| --- | --- | --- | --- | --- | --- |
| Education level | | | | | |
| HWD5 | < College | **1.223** | **1.096** | **1.364** | 0.239 |
|  | College | **1.094** | **1.002** | **1.194** |  |
|  | > College | 1.073 | 0.889 | 1.295 |  |
| HWD6 | < College | **1.210** | **1.052** | **1.391** | 0.700 |
|  | College | **1.125** | **1.005** | **1.260** |  |
|  | > College | 1.116 | 0.873 | 1.427 |  |
| HWD7 | < College | **1.197** | **1.039** | **1.379** | 0.731 |
|  | College | 1.114 | 0.997 | 1.245 |  |
|  | > College | 1.135 | 0.895 | 1.441 |  |
| HWD8 | < College | 1.127 | 0.922 | 1.377 | 0.337 |
|  | College | 0.934 | 0.786 | 1.110 |  |
|  | > College | 0.916 | 0.617 | 1.360 |  |
| HWD9 | < College | 1.282 | 0.990 | 1.661 | **0.050** |
|  | College | 0.852 | 0.657 | 1.106 |  |
|  | > College | 0.733 | 0.394 | 1.364 |  |
| HWD10 | < College | 1.074 | 0.804 | 1.435 | 0.794 |
|  | College | 0.997 | 0.785 | 1.265 |  |
|  | > College | 0.882 | 0.531 | 1.465 |  |
| HWD11 | < College | **1.670** | **1.162** | **2.400** | 0.192 |
|  | College | 1.076 | 0.769 | 1.505 |  |
|  | > College | 1.120 | 0.578 | 2.170 |  |
| HWD12 | < College | **1.791** | **1.122** | **2.857** | 0.061 |
|  | College | 0.756 | 0.437 | 1.307 |  |
|  | > College | 1.206 | 0.453 | 3.210 |  |
| Income level | | | | | |
| HWD1 | < 50^th^ | **1.128** | **1.073** | **1.186** | **0.036** |
|  | ≥ 50^th^ | 1.047 | 0.997 | 1.101 |  |
| HWD2 | < 50^th^ | **1.124** | **1.063** | **1.189** | 0.375 |
|  | ≥ 50^th^ | **1.085** | **1.027** | **1.147** |  |
| HWD3 | < 50^th^ | **1.123** | **1.053** | **1.198** | 0.977 |
|  | ≥ 50^th^ | **1.121** | **1.051** | **1.196** |  |
| HWD4 | < 50^th^ | **1.140** | **1.062** | **1.222** | 0.495 |
|  | ≥ 50^th^ | **1.101** | **1.024** | **1.183** |  |
| HWD5 | < 50^th^ | **1.126** | **1.030** | **1.231** | 0.995 |
|  | ≥ 50^th^ | **1.126** | **1.027** | **1.236** |  |
| HWD6 | < 50^th^ | 1.107 | 0.986 | 1.242 | 0.407 |
|  | ≥ 50^th^ | **1.187** | **1.053** | **1.337** |  |
| HWD7 | < 50^th^ | **1.142** | **1.020** | **1.278** | 0.902 |
|  | ≥ 50^th^ | **1.130** | **1.003** | **1.273** |  |
| HWD8 | < 50^th^ | 1.034 | 0.878 | 1.217 | 0.502 |
|  | ≥ 50^th^ | 0.949 | 0.787 | 1.146 |  |
| HWD9 | < 50^th^ | 1.020 | 0.813 | 1.280 | 0.734 |
|  | ≥ 50^th^ | 0.959 | 0.728 | 1.263 |  |
| HWD10 | < 50^th^ | 0.931 | 0.738 | 1.176 | 0.396 |
|  | ≥ 50^th^ | 1.082 | 0.837 | 1.398 |  |
| HWD11 | < 50^th^ | 1.103 | 0.816 | 1.492 | 0.179 |
|  | ≥ 50^th^ | 1.518 | 1.061 | 2.171 |  |
| HWD12 | < 50^th^ | 0.934 | 0.605 | 1.441 | 0.141 |
|  | ≥ 50^th^ | 1.542 | 0.925 | 2.572 |  |

Note: Bold font face indicates a statistically significant result (*P* < 0.05). The *P* value is for the interaction term between the heatwave exposure variable and the effect modifier.

^a^ No PROM case more than 35 years old experienced HWD12 in this study.

**Table S13.** The effect modification in associations between heatwaves and PROM by maternal characteristics estimated in models with the interaction term (continued).

| Description | | HRs | 95% CI | | *P* value for the interaction |
| --- | --- | --- | --- | --- | --- |
| Smoking | | | | | |
| HWD1 | Smoker | **1.131** | **1.040** | **1.229** | 0.303 |
|  | Non-smoker | **1.078** | **1.037** | **1.121** |  |
| HWD2 | Smoker | **1.173** | **1.069** | **1.287** | 0.163 |
|  | Non-smoker | **1.091** | **1.045** | **1.140** |  |
| HWD3 | Smoker | **1.169** | **1.049** | **1.303** | 0.425 |
|  | Non-smoker | **1.114** | **1.059** | **1.172** |  |
| HWD4 | Smoker | **1.232** | **1.095** | **1.387** | 0.083 |
|  | Non-smoker | **1.099** | **1.040** | **1.162** |  |
| HWD5 | Smoker | **1.294** | **1.113** | **1.504** | **0.047** |
|  | Non-smoker | **1.094** | **1.019** | **1.174** |  |
| HWD6 | Smoker | **1.414** | **1.169** | **1.712** | **0.016** |
|  | Non-smoker | 1.093 | 0.997 | 1.198 |  |
| HWD7 | Smoker | 1.162 | 0.948 | 1.424 | 0.809 |
|  | Non-smoker | **1.130** | **1.034** | **1.236** |  |
| HWD8 | Smoker | 1.130 | 0.829 | 1.540 | 0.377 |
|  | Non-smoker | 0.971 | 0.849 | 1.111 |  |
| HWD9 | Smoker | 1.095 | 0.697 | 1.722 | 0.635 |
|  | Non-smoker | 0.973 | 0.805 | 1.177 |  |
| HWD10 | Smoker | 1.101 | 0.692 | 1.753 | 0.639 |
|  | Non-smoker | 0.977 | 0.811 | 1.178 |  |
| HWD11 | Smoker | 0.893 | 0.401 | 1.993 | 0.394 |
|  | Non-smoker | **1.285** | **1.008** | **1.637** |  |
| HWD12 | Smoker | 0.971 | 0.314 | 3.006 | 0.796 |
|  | Non-smoker | 1.134 | 0.801 | 1.604 |  |

Note: Bold font face indicates a statistically significant result (*P* < 0.05). The *P* value is for the interaction term between the heatwave exposure variable and the effect modifier.

^a^ No PROM case more than 35 years old experienced HWD12 in this study.
